# Structures of multiple states of the nicotinamide nucleotide transhydrogenase from *Escherichia coli*

**DOI:** 10.1016/j.isci.2026.117657

**Published:** 2026-09-24

**Authors:** Jiao Li, Sangjin Hong, Rui Hao, Kai Zhang, Robert B. Gennis, Jiapeng Zhu

**Affiliations:** 1 Department of Cardiology, The First Affiliated Hospital of USTC, MOE Key Laboratory for Cellular Dynamics, Center for Advanced Interdisciplinary Science and Biomedicine of IHM, Division of Life Sciences and Medicine, Hefei National Research Center for Interdisciplinary Sciences at the Microscale, University of Science and Technology of China, Hefei 230026, China; 2 School of Medicine, Nanjing University of Chinese Medicine, Nanjing 210023, China; 3 Department of Biochemistry, University of Illinois at Urbana-Champaign, Urbana, IL 61801, USA; 4 Department of Hepatobiliary Surgery, The First Affiliated Hospital of Anhui Medical University, Hefei 230022, China

**Keywords:** nicotinamide nucleotide transhydrogenase, hydride transfer, proton pump, conformational dynamics, NADPH production

## Abstract

Nicotinamide nucleotide transhydrogenase couples hydride transfer between NAD(H) and NADP(H) to proton translocation, supporting cellular NADPH production. Here we present cryo-EM structures of full-length *Escherichia coli* transhydrogenase in apo, substrate-bound, and inhibitor-bound states. In the apo state, both copies of the NADP(H)-binding domain (dIII) adopt a face-down configuration, with the binding pocket partially embedded in transmembrane domain dII, plugging the cytoplasmic side of the proton channel. NADP^+^ or NADPH binding to dIII is accompanied by detachment of one or both dIII copies from dII and increased orientational dynamics. Detachment of dIII from dII allows a conformation compatible with hydride transfer between NADP(H) in dIII and NAD(H) in dI, and is accompanied by conformational changes in the dII proton channel that close periplasmic access and open cytoplasmic access. Palmitoyl-CoA stabilizes an apo-like conformation, trapping dIII in a face-down configuration. These results define the structural basis for transhydrogenase regulation through interdomain conformational coupling.

## Highlights

• Cryo-EM structures of full-length *E. coli* transhydrogenase in multiple states
• NADP(H) binding to dIII with dIII detachment and proton channel rearrangements
• Palmitoyl-CoA traps a closed apo-like state by binding the NADP(H) pocket
• Structures reveal the basis for transhydrogenase regulation and inhibition

## Graphical abstract

**Figure:**
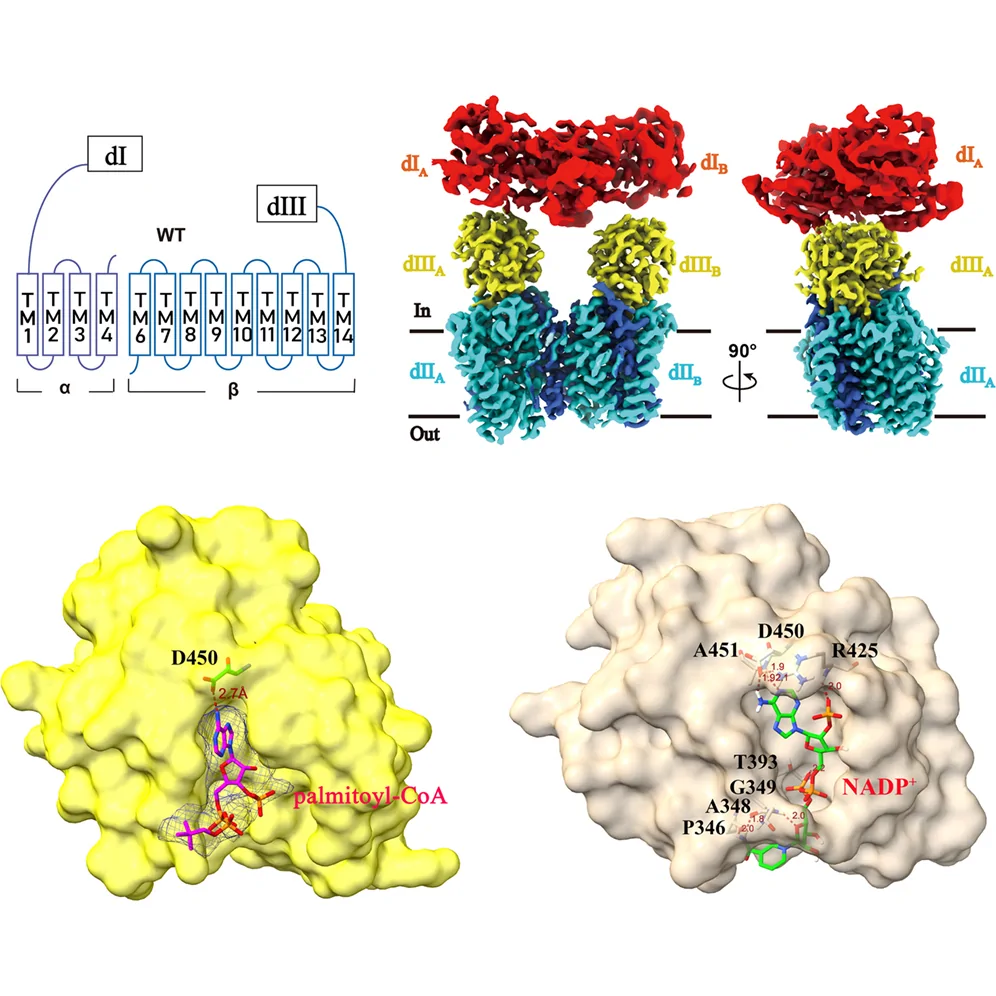

## Teaser

Biochemistry; Structural biology

## Introduction

Nicotinamide nucleotide transhydrogenase couples the proton motive force to the hydride transfer between NAD(H) and NADP(H).^1^^,^^2^^,^^3^^,^^4^ The enzyme spans the inner mitochondrial membrane of mammals and filamentous fungi, and the cytoplasmic membrane of a broad range of prokaryotes. For each hydride transfer, a proton is translocated electrogenically from the exterior (positive) to the interior (negative) side of the membrane, thereby supporting the generation of NADPH under most physiological conditions.

$$\text{NADH}+{\text{NADP}}^{+}+H_{\text{out}}^{+}⇄{\text{NAD}}^{+}+\text{NADPH}+H_{\text{in}}^{+}$$

In *E. coli*, transhydrogenase contributes approximately 40% of the cellular NADPH pool,^5^ and in mitochondria it also serves as a major source of NADPH together with other contributors such as isocitrate dehydrogenase, malic enzyme, and serine-dependent one-carbon metabolism.^6^^,^^7^^,^^8^^,^^9^ By sustaining the cellular NADPH pool, transhydrogenase plays a central metabolic role in linking catabolic and anabolic redox pathways and mitigating oxidative stress.

Disruption of mitochondrial redox balance has been linked to diverse pathophysiological conditions, and transhydrogenase is increasingly recognized as a key modulator of oxidative stress responses.^10^^,^^11^ Mutations in this enzyme cause familial glucocorticoid deficiency by impairing adrenal steroidogenesis,^12^ and altered transhydrogenase activity has been implicated in cancer biology. In non-small cell lung cancer, for example, transhydrogenase is frequently upregulated or post-translationally modified through interleukin-1β-induced acetylation to bolster NADPH production. This process mitigates oxidative stress and stabilizes iron-sulfur cluster-dependent enzymes, thereby protecting cancer cells from ferroptosis and promoting survival.^9^^,^^13^^,^^14^ The physiological consequences of transhydrogenase dysregulation are highly context-dependent; its silencing can confer chemoresistance, whereas its overexpression can suppress tumor growth, and its reversed activity in right ventricular failure leads to NADPH depletion and exacerbates oxidative damage.^15^^,^^16^

Transhydrogenase is a homodimer in which each protomer comprises three domains: domain I (dI), which binds NAD(H); domain III (dIII), which binds NADP(H); and the membrane-embedded domain II (dII), which houses a proton channel composed of 12–14 transmembrane helices (Figure 1A). Depending on the species, the protomer exists as a single subunit (e.g., mammalian), two subunits (e.g., *E. coli*), or three subunits (e.g., *Thermus thermophilus*).^1^^,^^3^^,^^17^ Structures of isolated recombinant domains have provided important insights into their organization and interactions.^1^^,^^18^^,^^19^^,^^20^^,^^21^^,^^22^^,^^23^^,^^24^^,^^25^^,^^26^^,^^27^ Isolated dI forms a stable dimer, (dI)_2_, whereas dIII is monomeric. When mixed, dI and dIII assemble into a heterotrimer consisting of two copies of dI and one copy of dIII, positioning NADP(H) bound to dIII adjacent to NAD(H) in one copy of dI. This arrangement enables direct hydride transfer from NADH to NADP^+^ without intermediate redox steps.^28^^,^^29^ The membrane domain dII also forms a dimer, and each copy contains a single protonatable residue and ordered water molecules that constitute the proton-conducting pathway.^23^^,^^27^

**Figure 1:**
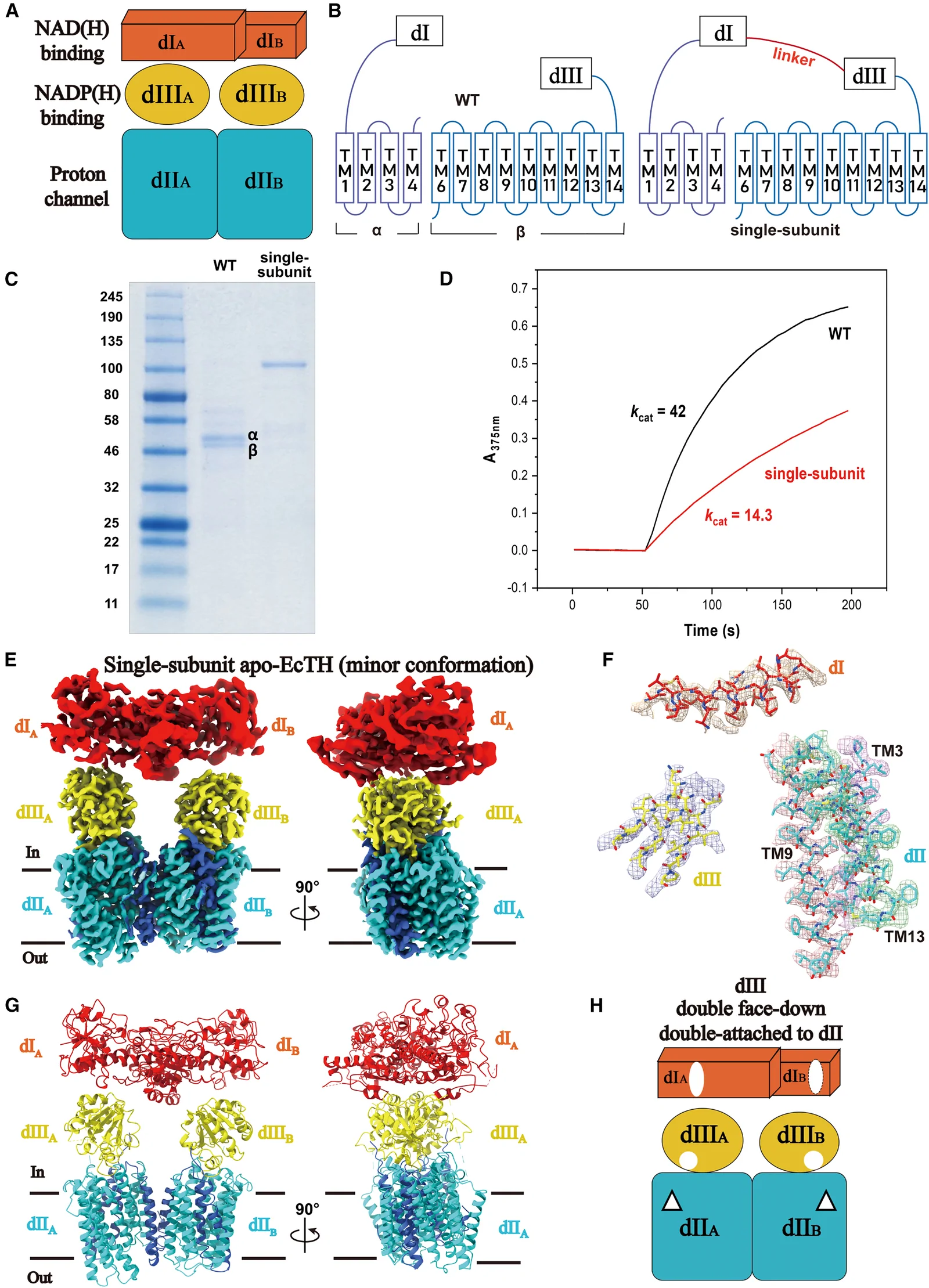
Domain organization and biochemical properties of EcTH, and the apo-state structure of the single-subunit enzyme (A) Schematic representation of EcTH, illustrating the domain organization, where dI harbors the NAD(H)-binding site, dIII the NADP(H)-binding site, and dII the proton channel. (B) Two-dimensional schematics of the α and β subunits of WT EcTH and the single-subunit EcTH variant. The α subunit comprises dI and the first four transmembrane helices (TM1-TM4) of dII, whereas the β subunit contributes the remaining nine transmembrane helices of dII (TM6-TM14) and all of dIII. To generate this construct mimicking protozoan transhydrogenases, β and α were fused using a linker sequence derived from the protozoan *Paratrimastix pyriformis* transhydrogenase (sequence provided in STAR Methods). (C) Expression and purification of WT and single-subunit EcTH analyzed by SDS-PAGE (4–20% gradient gel). (D) Reverse transhydrogenase activity assay. Reduction of the NAD^+^ analog APAD^+^ by NADPH was monitored at 375 nm and 37 °C (*ε* = 6.07 mM^−1^ cm^−1^); see STAR Methods for assay details. (E) Front and side views of the high-resolution cryo-EM map of the single-subunit apo-EcTH (minor conformation). (F) Representative densities showing high-resolution features of amino acid residues across the three domains. (G) Front and side views of the atomic model of the single-subunit apo-EcTH (minor conformation). (H) Schematic of the single-subunit apo-EcTH (minor conformation) showing both dIII copies in the face-down, double-attached conformation.

Structures of the entire transhydrogenase have been reported for *T. thermophilus*^17^ and ovine mitochondria,^30^ providing insights into how dIII repositions during catalysis. The low-resolution (∼7 Å) X-ray structure of *T. thermophilus* without bound ligands (apo-TtTH) revealed two distinct orientations of dIII: a face-down state in which the NADP(H)-binding site points toward the membrane domain dII and a face-up state oriented toward dI, consistent with alternating conformations proposed to support coupling between proton translocation and hydride transfer.^17^ Cryo-EM studies of ovine mitochondrial transhydrogenase (OmTH) demonstrated large ligand-dependent remodeling conformational changes.^30^ In the apo form (apo-OmTH), both copies of dIII adopt a face-down, “double-attached” conformation with the NADP(H)-binding site partially embedded in dII. NADP^+^-bound OmTH retains this predominant conformation but also contains a significant fraction of particles in which only one copy of dIII is visible while the second becomes highly dynamic. In contrast, NADPH binding induces global rearrangements in which dIII detaches from dII and samples multiple single- and double-detached conformations. However, no conformation displays a face-up orientation adjacent to an NAD(H)-binding site in (dI)_2_, a geometry compatible with direct hydride transfer between the dinucleotides. Detachment of dIII is accompanied by structural rearrangements in dII, including opening of the proton channel toward the matrix (N) side, closure toward the intermembrane-space (P) side, and reorientation of the key protonatable residue His664. These observations indicate that ligand-dependent positioning of dIII regulates access to the proton channel and shapes the catalytic cycle.

Although the structures of isolated (dI)_2_ and (dI)_2_(dIII) complexes of *E. coli* transhydrogenase (EcTH) have been reported,^21^^,^^31^ the architecture and conformational landscape of the full-length enzyme have remained unknown despite extensive biochemical investigation. Here, we present high-resolution cryo-EM structures of the entire *E. coli* enzyme under ligand-free, substrate-bound, and inhibitor-bound conditions, revealing previously unobserved conformational states. These structures also provide the first direct evidence for the basis of palmitoyl-CoA inhibition, showing that the inhibitor occupies the NADP(H)-binding pocket of dIII in a manner consistent with competitive inhibition.^32^^,^^33^

## Results

To increase the stability of EcTH, a single-subunit variant was genetically engineered by fusing the two native subunits, α (PntA) and β (PntB), with a linker joining the C-terminus of β to the N-terminus of α. This design mimics the natural single-polypeptide architecture of protozoan transhydrogenases, and the linker sequence was derived from the transhydrogenase of the protozoan *Paratrimastix pyriformis* (see STAR Methods).

Recombinant proteins with N-terminal His-tags were purified in LMNG detergent using immobilized metal-affinity chromatography followed by size-exclusion chromatography. The wild-type (WT) EcTH consists of two copies each of the α (54 kDa) and β (48 kDa) subunits, forming a dimer of α/β protomers (Figures 1B and 1C). In contrast, the single-subunit variant consists of two copies of a fused 102-kDa polypeptide, in which the sequences of α and β are joined in reverse order, with a linker sequence inserted between them (Figure 1C). Functional assays confirmed that the engineered enzyme retained catalytic activity, although its steady-state turnover rate (k_cat_) was reduced to 14.3 s^−1^, approximately 3-fold lower than that of WT EcTH (42 s^−1^; Figure 1D).

We examined EcTH under five conditions: (1) WT and single-subunit apo-EcTH in the absence of ligands; (2) WT EcTH in the presence of both NADP^+^ and NAD^+^; (3) WT and single-subunit EcTH in the presence of both NADPH and NADP^+^; (4) single-subunit EcTH in the presence of NADPH alone; and (5) single-subunit EcTH in the presence of palmitoyl-CoA. To facilitate navigation through the complex conformational landscape described later in discussion, we first summarize all conformational states observed under each condition in Table 1.

**Table 1:** Cryo-EM conformational states of WT and single-subunit EcTH under different ligand conditions

Sample	Conformation	dIII state	(dI)_2_ state	dII state	Key feature
WT apo	major	double attached, face-down	detached/weak	Closed (N-side)	no hydride transfer geometry
Single-subunit apo	minor	double attached, face-down	resolved	Closed (N-side)	full domain model
WT NADP^+^/NAD^+^	double attached	double attached, face-down	weak	Closed (N-side)	NADP^+^ in both dIII
WT NADP^+^/NAD^+^	single attached	one attached, one detached	tilted	Asymmetric	N-side channel opening on detached side
WT NADP^+^/NAD^+^	detached (dI)_2_	N/A	Resolved, NAD^+^ bound	N/A	focused refinement on (dI)_2_
WT NADPH/NADP^+^	single attached	one attached, one detached	tilted	asymmetric	similar to single attached (NADP^+^/NAD^+^)
WT NADPH/NADP^+^	double detached	both detached, one upward	associated	asymmetric	near hydride transfer geometry
Single-subunit NADPH	double attached	double attached, face-down	weak	closed (N-side), P-side constriction	NADPH-induced dII rearrangements
Single-subunit NADPH	single attached	one attached, one detached	tilted	asymmetric	similar to single attached
Single-subunit palmitoyl-CoA	major	double attached, face-down	detached/weak	closed (N-side)	palmitoyl-CoA in NADP(H)-binding pocket

### Apo-EcTH

A notable feature shared by both WT and single-subunit apo-EcTH is the face-down orientation of the NADP(H)-binding sites in two copies of domain III (dIII_A_ and dIII_B_) toward the membrane domain dII (Figures 1E and 1G). As a result, these domains adopt a face-down conformation, positioning their NADP(H)-binding sites far from the NAD(H)-binding sites in (dI)_2_ (Figure 1H). This architecture closely resembles the double-attached state previously described for apo-OmTH and places the NAD(H)- and NADP(H)-binding sites much too far apart to support hydride transfer.

For WT apo-EcTH, cryo-EM analysis reveals that the enzyme forms a dimer of α/β protomers (Figure S1). However, the density corresponding to the (dI)_2_ dimer is not resolved, indicating substantial conformational flexibility of this domain. Only domains dII and dIII are resolved, and the inter-domain linkers are not visible in any of the WT reconstructions.

The single-subunit apo-EcTH variant also forms a dimer (Figure S2). In the major conformation (80% of the particles), the (dI)_2_ dimer is not resolved. However, in a significant minor conformation (8% of the particles), the (dI)_2_ dimer is resolved (Figures 1E and 1F). The crystallographic structure of (dI)_2_ (PDB 1X13) was fitted into the corresponding density for the two sub-domains of dI (I_A_ and I_B_), followed by manual adjustment and real-space refinement using Coot to optimize the model.^34^ The resulting structure is consistent with the previously reported X-ray model of (dI)_2_.^21^

### Conformational changes in the presence of both NADP^+^ and NAD^+^

Incubation of purified, detergent-solubilized WT EcTH with 3 mM each of NADP^+^ and NAD^+^ yielded four distinct conformations: double dIIIs attached to dII, single dIII attached to dII, detached (dI)_2_, and a minor state with a well-resolved (dI)_2_ dimer (Figure S3).

#### Double dIIIs attached to dII (${\text{EcTH}}_{{{\text{NADP}}^{+}/\text{NAD}}^{+}}^{\text{double}\;\text{dIIIs}\;\text{attached}}$)

This state represents ∼26% of the dataset. Both dIII_A_ and dIII_B_ are resolved at 2.74 Å, each binding NADP^+^ in the face-down orientation (Figures 2A and S8A). As in apo-EcTH, the NAD(H)- and NADP(H)-binding sites are oriented away from each other, rendering the configuration incompatible with hydride transfer (Figure 2A). The (dI)_2_ dimer shows weak density, consistent with high mobility (Figures S3B and S3D). Upon NADP^+^ binding, loops C, D, and E surrounding the NADP(H)-binding pocket undergo remodeling that opens the pocket (Figure S9A and Video S1). Concomitantly, dII exhibits coordinated rearrangements. TM9, TM10, TM12, and TM13 shift outward, a movement more pronounced on the cytoplasmic than the periplasmic side (Figure 2E and Video S2). This displacement enlarges the hydrophilic pore formed by the conserved polar residues His91, Asn222, and Asn458, shifting the pore axis toward the positively charged periplasmic side (P side) and facilitating protonation of His91 (Figures S9D and S11B).

**Figure 2:**
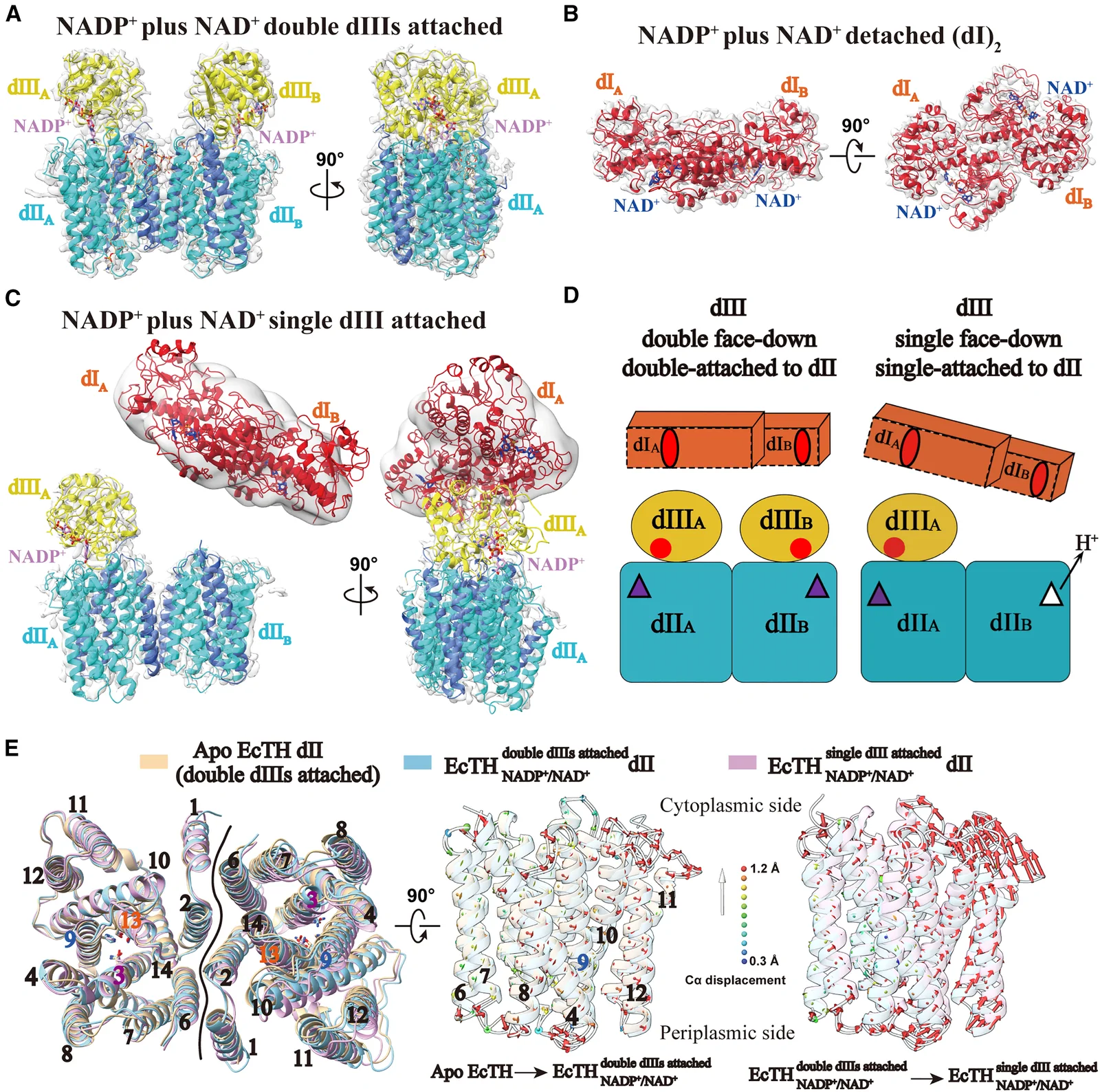
Conformational states of WT EcTH in the presence of both NADP^+^ and NAD^+^ (A) Structure of ${\text{WT}\;\text{EcTH}}_{{{\text{NADP}}^{+}/\text{NAD}}^{+}}^{\text{double}\;\text{dIIIs}\;\text{attached}}$. Both copies of dIII adopt a face-down orientation and remain attached to dII, with each dIII binding one NADP^+^ in its NADP(H)-binding site. The (dI)_2_ dimer exhibits substantial mobility, resulting in weak density that prevents determination of a complete (dI)_2_ structure. (B) Structure of ${\text{WT}\;\text{EcTH}}_{{{\text{NADP}}^{+}/\text{NAD}}^{+}}^{{\text{detached}\;\left(\text{dI}\right)}_{2}}$. When refinement was focused exclusively on the (dI)_2_ signal, this region displayed high-resolution features, with each dI protomer bound to one NAD^+^ at its NAD(H)-binding site. (C) Structure of ${\text{WT}\;\text{EcTH}}_{{{\text{NADP}}^{+}/\text{NAD}}^{+}}^{\text{single}\;\text{dIII}\;\text{attached}}$. One copy of dIII is completely detached from dII, while the other remains attached in a face-down orientation. The (dI)_2_ dimer is tilted toward the side where dIII is detached. (D) Schematic representations of ${\text{WT}\;\text{EcTH}}_{{{\text{NADP}}^{+}/\text{NAD}}^{+}}^{\text{double}\;\text{dIIIs}\;\text{attached}}$ and ${\text{WT}\;\text{EcTH}}_{{{\text{NADP}}^{+}/\text{NAD}}^{+}}^{\text{single}\;\text{dIII}\;\text{attached}}$. The red ellipse in (dI)_2_ represents the NAD^+^-bound state, and the red circle in dIII indicates the NADP^+^-bound state. Purple and white triangles denote protonated and deprotonated forms of His91, respectively, within the proton channel. (E) Conformational changes in the dII proton channel in the presence of NADP^+^ and NAD^+^. The dII domains from the ${\text{WT}\;\text{EcTH}}_{{{\text{NADP}}^{+}/\text{NAD}}^{+}}^{\text{double}\;\text{dIIIs}\;\text{attached}}$ and ${\text{WT}\;\text{EcTH}}_{{{\text{NADP}}^{+}/\text{NAD}}^{+}}^{\text{single}\;\text{dIII}\;\text{attached}}$ were aligned to the corresponding domain of the single-subunit apo-EcTH using the proton-channel helices TM3, TM9, and TM13 on one side. The overall superposition is viewed from the periplasmic side (left image). The conformational changes of the dII on the right side of the left image are shown separately: from apo to ${\text{WT}\;\text{EcTH}}_{{{\text{NADP}}^{+}/\text{NAD}}^{+}}^{\text{double}\;\text{dIIIs}\;\text{attached}}$ (middle image), and from ${\text{WT}\;\text{EcTH}}_{{{\text{NADP}}^{+}/\text{NAD}}^{+}}^{\text{double}\;\text{dIIIs}\;\text{attached}}$ to ${\text{WT}\;\text{EcTH}}_{{{\text{NADP}}^{+}/\text{NAD}}^{+}}^{\text{single}\;\text{dIII}\;\text{attached}}$ (right image). Displacement magnitudes are mapped onto Cα atoms and colored on a blue-to-red scale, representing shifts ranging from <0.3 Å (blue) to >1.2 Å (red).

Video S1. The conformational transition of dIII from the apo state to the NADP+-bound state

Video S2. The conformational transition of dII from the apo state to the double dIII-attached state (each dIII bound to NADP+)

#### Single dIII attached to dII (${\text{EcTH}}_{{{\text{NADP}}^{+}/\text{NAD}}^{+}}^{\text{single}\;\text{dIII}\;\text{attached}}$)

This conformation represents approximately 28% of the dataset. In this state, the density for the (dI)_2_ dimer remains weaker than that for dII and dIII (Figures S3B and S3D), allowing reliable modeling only of the attached dIII and dII domains (Figure 2C). Although the (dI)_2_ density is poorly resolved, its overall dimeric outline could be tentatively fitted (Figure 2C). One copy of dIII is completely detached from dII, accompanied by a tilt of the (dI)_2_ dimer toward the same side (Figures 2C, 2D, S3B, and S3D). The second copy of dIII remains face-down and attached to the membrane domain dII, like the configuration observed in ${\text{WT}\;\text{EcTH}}_{{{\text{NADP}}^{+}/\text{NAD}}^{+}}^{\text{double}\;\text{dIIIs}\;\text{attached}}$ (Figure 2A), and binds NADP^+^ in its NADP(H)-binding site (Figure S8B).

Compared with apo-EcTH and the double dIII-attached state, domain II in the single dIII-attached conformation undergoes more pronounced and asymmetric rearrangements. On the dIII-attached side, the proton channel helices retain an apo-like alignment. In contrast, on the dIII-detached side, multiple transmembrane helices, including TM3, TM4, TM7, and TM9-13, shift outward toward the cytoplasmic surface, with greater displacements occurring toward the periplasmic side in the regions of TM3, TM9, and TM13 (Figure 2E and Video S3). These movements shift the hydrophilic pore formed by His91, Asn222, and Asn458 toward the negatively charged cytoplasmic (N) side and increase its diameter by approximately 2 Å (Figures 2E, S9D, and S11D). This asymmetry is consistent with the positional differences of the dII helices comparing structures of dII in which dIII is attached or detached (Figures S10B and S10C) and appears optimized to (*1*) facilitate proton release from protonated His91 into the cytoplasm during the forward reaction, thereby promoting dIII dissociation from dII, and (*2*) prime the periplasmic side for subsequent opening of the proton channel.

Video S3. The conformational transition of dII from the double dIII-attached state (each dIII bound to NADP+) to the single dIII-attached state

#### Detached (dI)_2_ (${\text{EcTH}}_{{{\text{NADP}}^{+}/\text{NAD}}^{+}}^{{\text{detached}\;\left(\text{dI}\right)}_{2}}$)

When refinement was focused on the dI domain, a well-defined (dI)_2_ dimer was reconstructed at 3.38 Å resolution, with each protomer bound to NAD^+^ (Figures 2B, S3B, and S3D). This clearly demonstrates that both NAD(H)-binding sites are occupied under these conditions.

#### Minor well-resolved (dI)_2_ state (${\text{EcTH}}_{{{\text{NADP}}^{+}/\text{NAD}}^{+}}^{\text{minor}}$)

A low-population conformation was also identified in which the (dI)_2_ dimer is more clearly resolved in the density map. Although not abundant enough to quantify, this state further supports dynamic variability of the dI-dII-dIII assembly.

### Conformational changes in the presence of both NADPH and NADP^+^

Purified, detergent-solubilized WT EcTH and the single-subunit variant were incubated with 3 mM each of NADPH and NADP^+^ prior to cryo-EM grid preparation. In addition to the conformations observed in the absence of ligands, two additional states, single dIII attached to dII and double dIIIs detached from dII, were identified in both datasets.

#### Single dIII attached to dII (${\text{EcTH}}_{{\text{NADPH}/\text{NADP}}^{+}}^{\text{single}\;\text{dIII}\;\text{attached}}$)

This conformation accounts for ∼18% of WT particles and ∼23% of single-subunit particles. Because of weak densities for (dI)_2_, dII, and dIII, the corresponding domains from the apo-EcTH structure were docked to obtain a fitted molecular model (Figure 3A). The resulting architecture closely resembles the previously described ${\text{WT}\;\text{EcTH}}_{{{\text{NADP}}^{+}/\text{NAD}}^{+}}^{\text{single}\;\text{dIII}\;\text{attached}}$ conformation. One copy of dIII is completely detached from dII and is accompanied by a tilt of the (dI)_2_ dimer toward the same side (Figures 3A, 3B, S4B, and S4E). The detached dIII further associates with neighboring particles to form a trimeric assembly, interpreted as a cryo-EM artifact not observed in the apo-EcTH dataset. This conformation is depicted as ${\text{EcTH}}_{{\text{NADPH}/\text{NADP}}^{+}}^{\text{detached}\;\text{dIII}}$ in Figures S4B and S4E. The second copy of dIII remains attached to dII in a face-down orientation, such as ${\text{WT}\;\text{EcTH}}_{{{\text{NADP}}^{+}/\text{NAD}}^{+}}^{\text{single}\;\text{dIII}\;\text{attached}}$ (Figure 2C).

**Figure 3:**
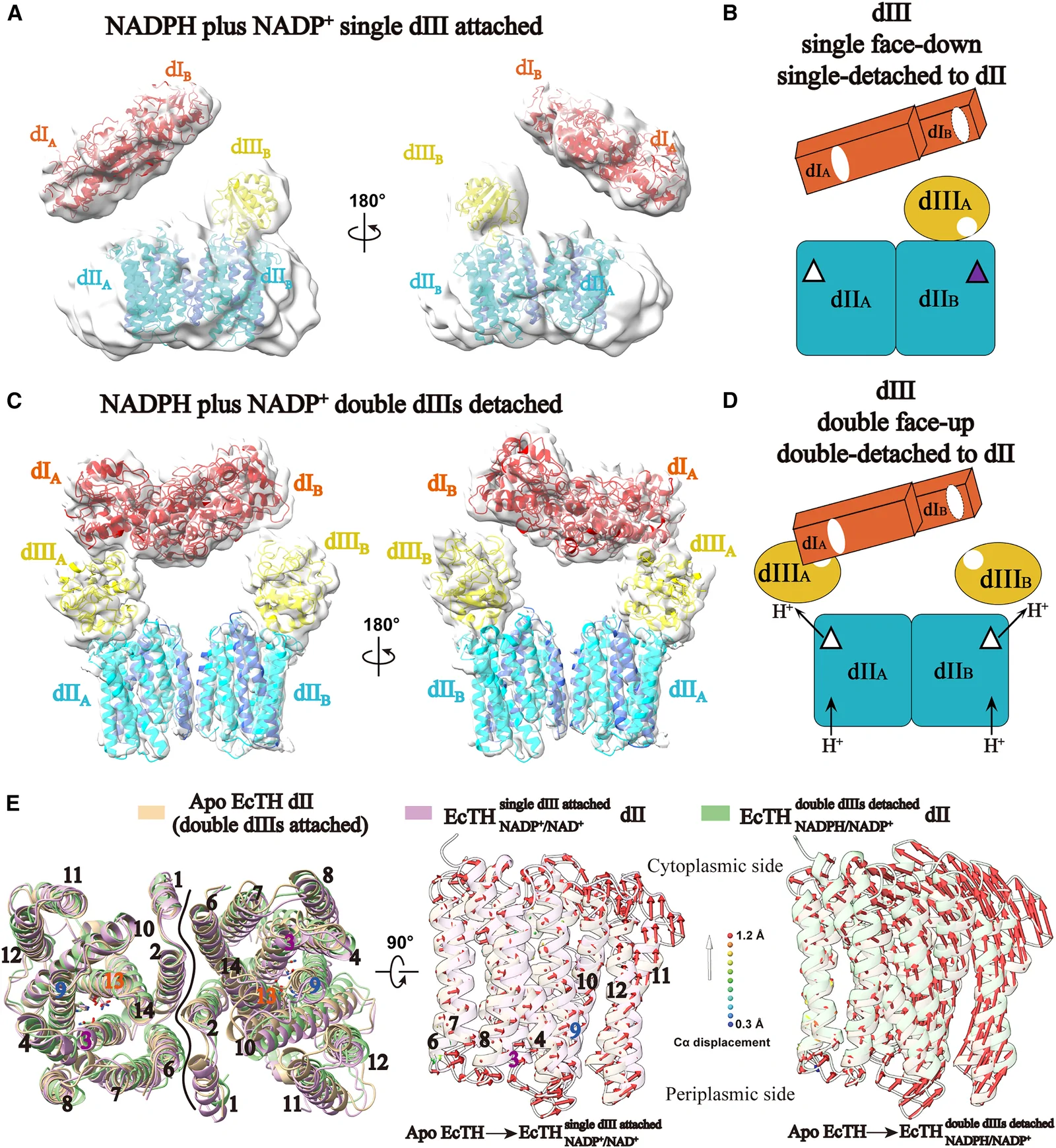
Conformational states of EcTH in the presence of both NADPH and NADP^+^ (A) Structure of ${\text{EcTH}}_{{\text{NADPH}/\text{NADP}}^{+}}^{\text{single}\;\text{dIII}\;\text{attached}}$. One copy of dIII is completely detached from dII, as reflected by the absence of density after global alignment. The other copy of dIII remains visible in a face-down orientation and attached to dII. The (dI)_2_ dimer tilts toward the side where dIII is detached. (B) Schematic representation of ${\text{EcTH}}_{{\text{NADPH}/\text{NADP}}^{+}}^{\text{single}\;\text{dIII}\;\text{attached}}$. Purple and white triangles represent protonated and deprotonated His91, respectively, in the proton channel. (C and D) Structure of ${\text{EcTH}}_{{\text{NADPH}/\text{NADP}}^{+}}^{\text{double}\;\text{dIIIs}\;\text{detached}}$ and the corresponding schematic representation. The white triangle denotes deprotonated His91 in the proton channel. (E) Conformational changes of the transmembrane helices in dII upon detachment of one or both dIII domains. The dII domains from the dIII-attached side of ${\text{WT}\;\text{EcTH}}_{{{\text{NADP}}^{+}/\text{NAD}}^{+}}^{\text{single}\;\text{dIII}\;\text{attached}}$ and from the dI-dIII interface-forming side of ${\text{EcTH}}_{{\text{NADPH}/\text{NADP}}^{+}}^{\text{double}\;\text{dIIIs}\;\text{detached}}$ were superimposed onto the corresponding domain of single-subunit apo-EcTH, viewed from the periplasmic side (left image), using TM3, TM9, and TM13 on the left-side proton channel as the alignment reference. The conformational changes in the right-side dII domain are shown separately: the dII transitions from apo to ${\text{WT}\;\text{EcTH}}_{{{\text{NADP}}^{+}/\text{NAD}}^{+}}^{\text{single}\;\text{dIII}\;\text{attached}}$ (middle image) and to ${\text{EcTH}}_{{\text{NADPH}/\text{NADP}}^{+}}^{\text{double}\;\text{dIIIs}\;\text{detached}}$ (right image), respectively. Displacement magnitudes are colored as in Figure 2E.

#### Double dIIIs detached (${\text{EcTH}}_{{\text{NADPH}/\text{NADP}}^{+}}^{\text{double}\;\text{dIIIs}\;\text{detached}}$)

This conformation accounts for ∼20% of the WT particles and ∼11% of single-subunit variant particles. One of the NAD(H)-binding sites in (dI)_2_ is oriented toward the upward-facing NADP(H)-binding pocket of one detached dIII (Figure 3C), although the separation remains too large to support hydride transfer. The densities for (dI)_2_ and both dIII copies are weaker than for dII, consistent with increased mobility. Thus, apo-derived models were docked to the corresponding densities (Figure 3C).

In this state, both dIII_A_ and dIII_B_ are detached from their membrane domains (dII_A_ and dII_B_), and each dIII is rotated such that its NADP(H)-binding site faces upward toward (dI)_2_ (Figures 3C and 3D). Domain dI_A_ associates with dIII_A_ in a configuration resembling an intermediate between the apo state and the soluble (dI)_2_(dIII) heterotrimer (PDB 2BRU), in which NADH (in dI_A_) and NADP^+^ (in dIII_A_) are oriented for hydride transfer.

Detachment of dIII_B_ from dII_B_, and the lack of a corresponding dI-dIII interface in this protomer, is accompanied by more pronounced rearrangements in dII_B_ (Figure 3E and Video S4). TM3 (specifically in the segment above its 3_10_-helix stretch^30^), TM4, TM9, TM11, and TM13 undergo longitudinal shifts toward the cytoplasmic surface (Figures 3E and S10D). This movement repositions His91 (on TM9) toward both the center of the proton channel and the cytoplasmic side (Figures S11G and S11H), a shift that likely facilitates proton release into the cytoplasm.

Video S4. The conformational transition of dII from the single dIII-attached state to the double dIII-detached state

Following this rearrangement, the proton translocation pathways of the two dII domains exhibit similar overall geometries except for a vertical offset, with the channel lacking a dIII-dI interface positioned closer to the cytoplasmic surface (Figure S10D). Both proton channels constrict to form a narrow region at the center of the proton channel at the cytoplasmic side (Figures S10D and S12E).

In contrast to the symmetric dII domains observed in both the apo and double dIII-attached states (${\text{WT}\;\text{EcTH}}_{{{\text{NADP}}^{+}/\text{NAD}}^{+}}^{\text{double}\;\text{dIIIs}\;\text{attached}}$ above, ${\text{EcTH}}_{\text{NADPH}}^{\text{double}\;\text{dIIIs}\;\text{attached}}$ and ${\text{EcTH}}_{\text{palmitoyl}-\text{CoA}}^{\text{major}}$ below), the ${\text{EcTH}}_{{\text{NADPH}/\text{NADP}}^{+}}^{\text{double}\;\text{dIIIs}\;\text{detached}}$ conformation exhibits marked asymmetry between its two dII domains (Figure S10). The dII domain lacking a dIII-dI interface undergoes a larger cytoplasmic displacement than its interface-forming counterpart, consistent with the trend seen in the ${\text{EcTH}}_{{\text{NADPH}/\text{NADP}}^{+}}^{\text{single}\;\text{dIII}\;\text{attached}}$ conformation.

### Conformational changes in the presence of NADPH

Three distinct conformations of the single-subunit EcTH were observed in the presence of 3 mM NADPH: one with both dIIIs attached to the membrane domain dII, one with only a single dIII attached to dII with the other detached, and a minor conformation with diffuse density across all domains (Figure S5).

#### Double dIIIs attached to dII (${\text{EcTH}}_{\text{NADPH}}^{\text{double}\;\text{dIIIs}\;\text{attached}}$)

This conformation represents approximately 37% of the particles. Both dII and dIII exhibit well-defined densities (Figure S5C), with both copies of dIII in the face-down orientation and attached to the membrane domain dII. Each dIII binds one NADPH (Figures S6A and S8C). NADPH binding induces remodeling of loops C, D, and E and their flanking secondary structures, most notably helix 3, leading to expansion of the NADP(H)-binding pocket with a defined outward displacement vector (Figure S9B and Video S5).

Video S5. The conformational transition of dIII from the apo state to the NADPH-bound state

The density for dI is faintly visible in 2D class averages (Figure S5B) but too weak to be resolved in the reconstructed 3D map. Binding of NADPH to dIII in the double-attached state results in conformational changes in dII compared to the apo state of the enzyme (which also has both dIII domains attached to dII). The NADPH-induced conformational changes in dII in the double-attached state are distinct from the conformational changes induced by the binding of NADP^+^ to dIII, though in both cases the proton channel remains closed on the cytoplasmic side since the attached dIII acts as a plug. Transmembrane helices TM3, TM4, TM6, TM7, and TM8 (Figure S6E), as well as TM9, show a shift toward the periplasmic side by about 1 Å (Figure S11F). As a consequence, His91 (on TM9) becomes displaced toward the periplasmic side relative to the apo state. In addition, transmembrane helices TM3 and TM13 move to form a constriction on the periplasmic side of the proton channel (Figures S6E and S12F and Videos S6 and S7).

Video S6. The conformational transition of dII from the double dIII-detached state to the double dIII-attached state (each dIII bound to NADPH)

Video S7. The dynamic cycle of dII The video shows the progression from the apo state through the double dIII-attached state (each dIII bound to NADP^+^), the single dIII-attached state, the double dIII-detached state, and the double dIII-re-attached state (each dIII bound to NADPH), returning to the initial apo state and completing the cycle. Structures were aligned using one side of the proton channel helices (TM3, TM9, and TM13). For details, see Figures 2E, 3E, 4E, and S7E. The dynamic cycle of the dII dimer is visualized from multiple perspectives, including top, bottom, and side views. Apo-EcTH, ${\text{EcTH}}_{{{\text{NADP}}^{+}/\text{NAD}}^{+}}^{\text{double}\;\text{dIIIs}\;\text{attached}}$, ${\text{EcTH}}_{{{\text{NADP}}^{+}/\text{NAD}}^{+}}^{\text{single}\;\text{dIII}\;\text{attached}}$, ${\text{EcTH}}_{{\text{NADPH}/\text{NADP}}^{+}}^{\text{double}\;\text{dIIIs}\;\text{detached}}$, and ${\text{EcTH}}_{\text{NADPH}}^{\text{double}\;\text{dIIIs}\;\text{attached}}$ are shown in light brown, cyan, violet, light green, and deep green cartoon representations, respectively, as in Figures 2E, 3E, 4E and S7E.

#### Single dIII attached to dII (${\text{EcTH}}_{\text{NADPH}\;}^{\text{single}\;\text{dIII}\;\text{attached}}$)

This conformation accounts for approximately 16% of the particles. One copy of dIII is completely detached and not visible after global alignment, while the density for (dI)_2_ is weak and tilts toward the detached side (Figure S6C). The overall domain arrangement resembles the structures of ${\text{WT}\;\text{EcTH}}_{{{\text{NADP}}^{+}/\text{NAD}}^{+}}^{\text{single}\;\text{dIII}\;\text{attached}}$ (Figure 2C) and ${\text{EcTH}}_{{\text{NADPH}/\text{NADP}}^{+}}^{\text{single}\;\text{dIII}\;\text{attached}}$ (Figure 3A).

#### Minor NADPH-bound conformation (${\text{EcTH}}_{\text{NADPH}}^{\text{minor}}$)

This conformation represents approximately 16% of the particles and exhibits density across all domains, but the resolution is insufficient for molecular modeling (Figure S5C). Its overall architecture resembles that of the single-subunit apo-EcTH minor conformation (Figure 1E), with both copies of dIII oriented face-down and attached to the membrane domain dII.

### Conformational changes in the presence of palmitoyl-CoA

Previous studies have shown that palmitoyl-CoA selectively competes with NADP(H), thereby blocking hydride transfer between NADP(H) and NAD(H).^33^ In this study, the single-subunit EcTH was incubated with 1 mM palmitoyl-CoA prior to cryo-EM grid preparation. Two distinct conformational states of the single-subunit EcTH were observed in the presence of palmitoyl-CoA (Figure S7).

#### Double dIIIs attached to dII with unresolved (dI)_2_ (${\text{EcTH}}_{\text{palmitoyl}-\text{CoA}}^{\text{major}}$)

This conformation represents approximately 49% of the dataset. Both copies of dIII are visible in the face-down orientation and attached to dII (Figures 4A, 4B, and S7). The density for the (dI)_2_ dimer is extremely weak and essentially undetectable. Domains II and III are resolved at 2.97 Å, and the density map for the adenosine-3′-phosphate (Ade) and pyrophosphate (Pyr) moieties of palmitoyl-CoA is well defined (Figures 4A, 4C, and S8D). The N6 atom of the purine ring in palmitoyl-CoA forms a hydrogen bond with the OD1 atom of Asp450, located near the NADP(H)-binding site of dIII (Figure 4C). Palmitoyl-CoA is buried within the pocket, partially occupying the position normally taken by NADP(H). Notably, palmitoyl-CoA binding induces only minor rearrangements within the dIII binding pocket compared to NADP(H) binding (Figure S9C).

**Figure 4:**
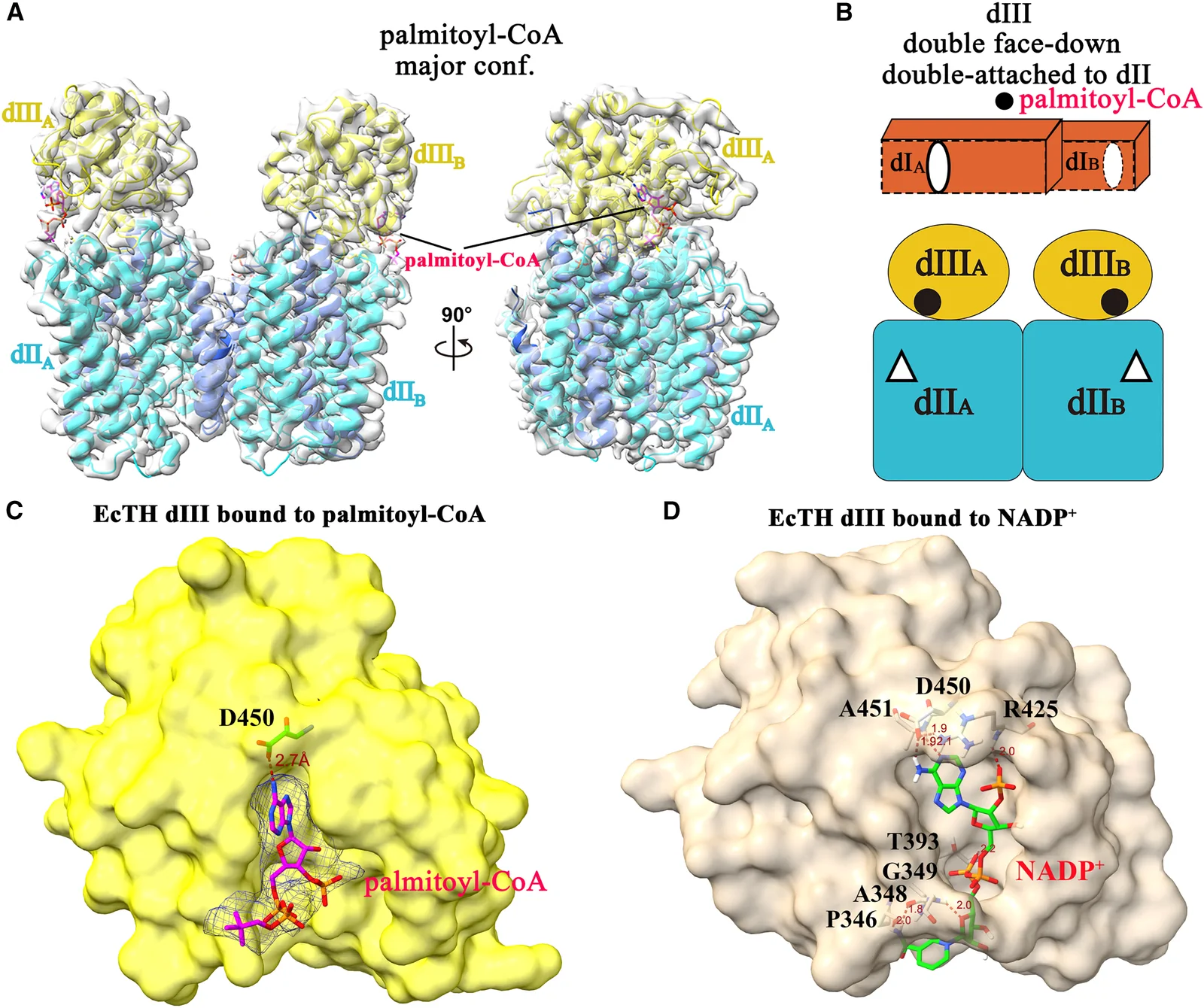
Major conformation of EcTH in the presence of palmitoyl-CoA (A) Structure of ${\text{EcTH}}_{\text{palmitoyl}-\text{CoA}}^{\text{major}}$. The density for the (dI)_2_ dimer is extremely weak and essentially undetectable. Both copies of dIII adopt a face-down orientation and remain attached to the membrane domain dII. Palmitoyl-CoA is buried within the NADP(H)-binding site. (B) Schematic representation of ${\text{EcTH}}_{\text{palmitoyl}-\text{CoA}}^{\text{major}}$. (C) Binding site of palmitoyl-CoA in ${\text{EcTH}}_{\text{palmitoyl}-\text{CoA}}^{\text{major}}$. One copy of dIII is shown as a yellow surface, and palmitoyl-CoA is depicted as magenta sticks. The density map for palmitoyl-CoA is contoured in medium blue. The side chain of Asp450, located near palmitoyl-CoA, is shown as green sticks and labeled. A hydrogen bond between the N6 atom of palmitoyl-CoA and OD1 atom of Asp450 is indicated with red dashed lines, and the distance is labeled in red (Å). (D) NADP(H)-binding site in the solution NMR structure of the soluble (dI)_2_(dIII) heterotrimer (PDB 2BRU). In this structure, dIII is oriented face-up and positioned adjacent to the (dI)_2_ dimer, shown as a light brown surface. NADP^+^ is depicted as green sticks. Side chains of residues near NADP^+^ are shown as gray sticks and labeled. Hydrogen bonds are indicated by red dashed lines, and distances are labeled in red (Å).

For comparison, Figure 4D shows NADP(H) bound to dIII in the solution NMR structure of the soluble (dI)_2_(dIII) heterotrimer (PDB 2BRU), where dIII adopts a face-up orientation, and the bound NADP^+^ is adjacent to one of the NAD(H)-binding sites in the (dI)_2_ dimer. In contrast, in the major palmitoyl-CoA-bound state, dIII is face-down, and the bound palmitoyl-CoA lies adjacent to the membrane domain dII. Many residues in dIII that contact NADP^+^ in the (dI)_2_(dIII) heterotrimer, Pro346, Ala348, Gly349, Thr393, Ala451, and Arg425, do not interact with palmitoyl-CoA in this structure (Figure 4D). The dII conformation is identical to that in the single-subunit apo-EcTH (Figure S9F).

#### Double dIIIs attached to dII with visible (dI)_2_ (${\text{EcTH}}_{\text{palmitoyl}-\text{CoA}}^{\text{minor}}$)

This conformation represents approximately 23% of the dataset (Figure S7C). It closely resembles the ${\text{EcTH}}_{\text{palmitoyl}-\text{CoA}}^{\text{major}}$ conformation, with both copies of dIII in the face-down orientation and attached to dII. Each dIII is bound to palmitoyl-CoA at the NADP(H)-binding site, as observed in the major state. The density for (dI)_2_ is better resolved and can be modeled in a manner like that of the single-subunit apo-EcTH structure (Figure 1E).

## Discussion

The structural study of OmTH demonstrated that the enzyme undergoes global conformational changes in response to the binding of NADPH or NADP^+^ to dIII,^30^ consistent with earlier biochemical studies.^35^^,^^36^ The current work extends these findings by examining EcTH under apo conditions, with NADP^+^ plus NAD^+^, NADPH plus NADP^+^, NADPH alone, and with the competitive inhibitor palmitoyl-CoA. All structures were determined at pH 8, and the protonation state of His91 within the proton channel cannot be directly inferred from the cryo-EM maps. Likewise, in the structures obtained with both NADPH and NADP^+^, the two ligands cannot be distinguished in the dIII binding pocket.

As in OmTH, both dIII and the (dI)_2_ dimer in EcTH exhibit substantial conformational flexibility, often resulting in weak or discontinuous densities. This motivated the design of a single-subunit EcTH variant, in which the native α and β subunits were fused in reverse order using a linker derived from the protozoan *Paratrimastix pyriformis* transhydrogenase. This construct directly links dIII to dI and retains approximately one-third of WT activity. In contrast to WT, the (dI)_2_ density in the single-subunit apo structure is sufficiently well resolved to enable modeling of all three domains (Figure 1E). The structural model of the (dI)_2_ dimer was generated by docking the previously determined X-ray structure of (dI)_2_ (PDB 1X13)^21^ into the cryo-EM density map of single-subunit apo-EcTH (Figure 1F), followed by real-space refinement using Coot. The resulting model closely resembles the (dI)_2_ structure from the X-ray study (PDB 1X13).

It is important to note, however, that because the chimera is an engineered construct with an artificial linker, it may not fully recapitulate the native conformational dynamics of WT EcTH, particularly as the linker could potentially restrict or bias the movements of dIII relative to dI. Although the major ligand-dependent conformational transitions observed in the chimera, including apo double-attached, single-detached, and double-detached states, were also observed in the WT enzyme, the structural data from the chimera are best viewed as complementing, rather than replacing, the native WT structures. Future studies using proteoliposome reconstitution under a proton gradient will be required to directly assess the coupling efficiency of the chimera in the forward direction.

Despite these considerations, the essential features of the single-subunit apo-EcTH are consistent with those of the OmTH structure in the absence of ligands. Both copies of dIII are attached to the membrane domain (dII) and adopt a face-down orientation, with the NADP(H)-binding sites facing away from (dI)_2_, an arrangement incompatible with direct hydride transfer with NAD(H). Across the ligand-bound datasets, the (dI)_2_ density is weaker than that of dII and dIII after global alignment, but docking the apo-derived (dI)_2_ into these maps reliably captures its domain-level orientation. Meanwhile, dIII adopts different orientations across ligand states due to its partial or complete detachment from dII. These observations indicate that (dI)_2_ and dIII move largely as independent rigid bodies, and that their relative orientations differ among states. Our structures provide direct evidence that nucleotide binding to dIII reshapes the conformational landscape of EcTH, inducing partial or complete detachment of one or both dIII domains and large-scale rearrangements in the transmembrane helices of dII, including state-specific movements around His91. Together, these coordinated changes indicate that nucleotide binding, domain detachment, and proton-channel gating are closely coupled during the catalytic cycle.

The major results from this study can be summarized as follows: (1) Binding of either NADP^+^ or NADPH to dIII leads to dIII detachment from dII in a subset of particles, yielding both single-detached and double-detached conformations. (2) Detachment of dIII is associated with rearrangements in the transmembrane helices that form the proton channel, opening toward the cytoplasmic side and closing it on the periplasmic side. (3) Even in the double-attached state with NADPH, dII displays rearrangements distinct from those in the apo and NADP^+^-bound states, although the cytoplasmic end of the channel remains closed by the attached dIII in all of them. (4) In the double-detached state, a conformation of EcTH is observed in which one NAD(H)-binding site in (dI)_2_ is oriented toward the NADP(H)-binding site in one dIII that remains associated with the (dI)_2_ dimer, distinguishing it from the second, more distal dIII. Although the distance remains too long to support direct hydride transfer, this upward-facing, (dI)_2_-associated arrangement resembles an intermediate geometry that links the membrane-bound face-down conformation to the soluble (dI)_2_·dIII heterotrimer determined by NMR (PDB 2BRU). (5) The structures further indicate that two detached dIII domains do not engage (dI)_2_ simultaneously; only one adopts a (dI)_2_-associated orientation while the second dIII remains distal. This asymmetry is consistent with hydride transfer proceeding sequentially in the two-halves of the dimer in alternating conformational states. (6) Palmitoyl-CoA binds within the NADP(H)-binding pocket of dIII, partially overlapping with the NADP(H)-binding site and sterically blocking NADP(H) access. This provides the structural basis for the competitive inhibition of NADP(H) binding by palmitoyl-CoA reported in earlier biochemical studies.^32^^,^^33^

These structural observations from the single-subunit EcTH chimera form the basis for an updated mechanistic model of its catalytic cycle (Figure 5). Because linker topology differs among TH families, the model is most directly applicable to THs in which (dI)_2_ remains connected to the rest of the enzyme through either dI-dII or dI-dIII linkers. In contrast, three-subunit THs such as that from *T. thermophilus*, which lack such covalent connectivity between (dI)_2_ and the rest of the enzyme, may require anti-phase dIII movements to maintain association of the (dI)_2_ dimer with the dII/dIII-containing complex. This is supported by the observation that the structure of the apo form of the *T. thermophilus* TH has one copy of dIII in a face-up conformation. At all times, (dI)_2_ must remain bound to one of the copies of dIII.

**Figure 5:**
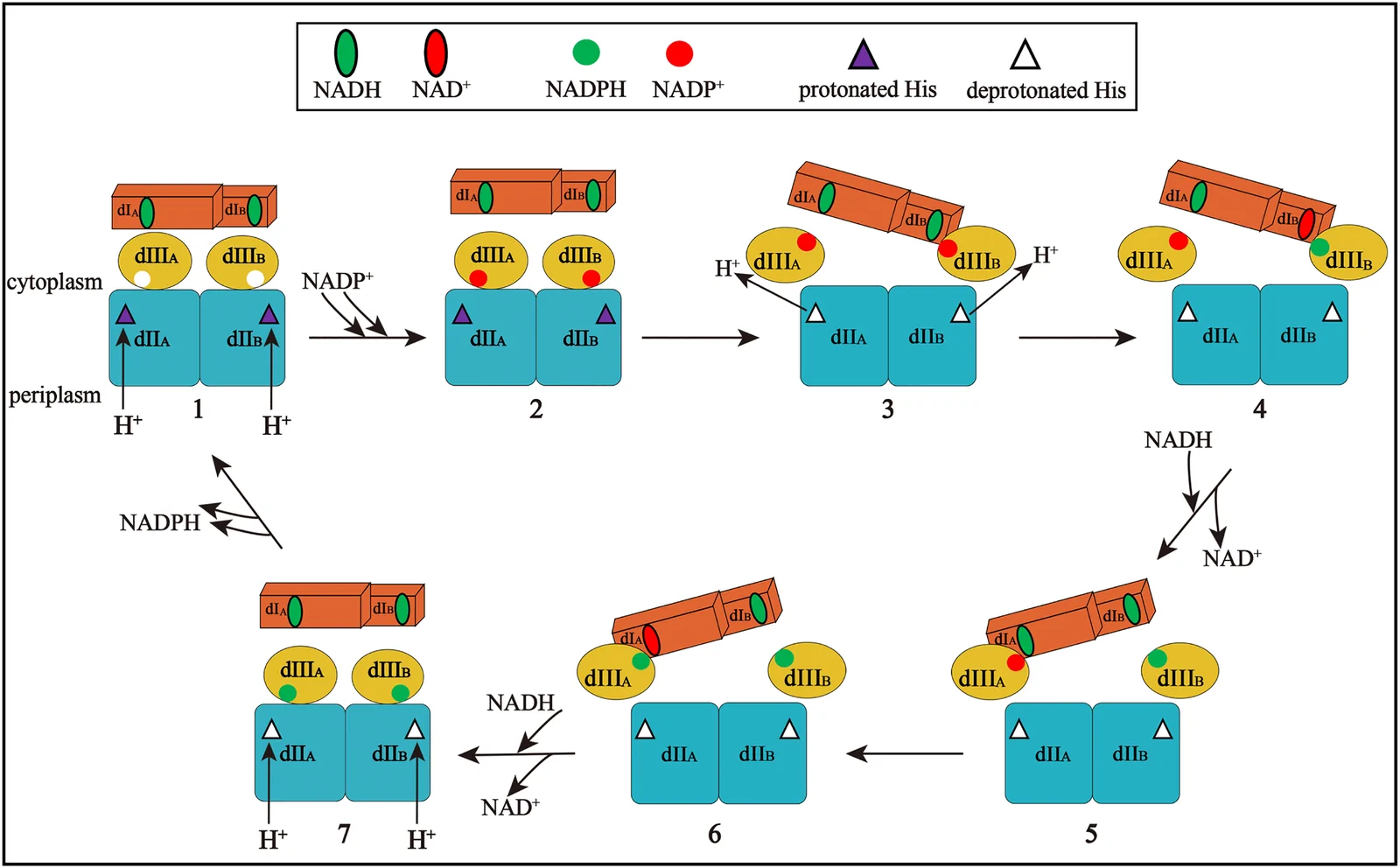
Proposed catalytic cycle of the single-subunit EcTH chimera Green and red ellipses in (dI)_2_ represent the NADH-bound and NAD^+^-bound states, respectively. Green and red circles in dIII denote the NADPH-bound and NADP^+^-bound states, respectively. Purple and white triangles indicate the protonated and deprotonated forms of His91, respectively, in the proton channel. The NAD(H)-binding sites in dI (ellipses) are assumed to exchange rapidly with NAD^+^ and NADH.

In the model shown in Figure 5, the proton-motive force, along with mass action due to substrate concentrations, provides the thermodynamic driving force for forward NADPH production. Each copy of dIII must undergo alternating conformations between face-up and face-down orientations. Substrate/product exchange can only occur with dIII in the face-down, attached conformation, whereas hydride transfer can only occur with dIII in the face-up, detached orientation. The overall framework of the model in Figure 5 aligns well with concepts introduced in previous models,^30^^,^^37^ and the present work refines and extends these proposals by incorporating the conformational states revealed in our cryo-EM structures. For clarity, the reaction is considered under conditions favoring the forward direction of catalysis (i.e., NADPH generation), with the following assumptions: a proton-motive force is positive on the periplasmic side, [NADH] >> [NAD^+^], and [NADP^+^] >> [NADPH]. In this model, when dIII is attached to the membrane domain dII, the proton channel is sealed at the cytoplasmic end, and the NADP(H)-binding site in dIII rapidly exchanges its bound dinucleotide with the cytoplasmic pool. Detachment of dIII from dII opens the channel on the cytoplasmic side while closing it on the periplasmic side. In this detached state, NADP(H) bound to dIII becomes occluded, preventing dissociation. The model is summarized later in discussion.

(1) The binding and release of NADH and NAD^+^ at binding sites in the (dI)_2_ dimer are rapid processes that occur independently of global conformational changes. Under conditions where [NADH] >> [NAD^+^], the product of the forward reaction, NAD^+^, rapidly dissociates and is replaced by NADH (steps 4→5 and 6→7 in Figure 5).
(2) When the NADP(H)-binding site in dIII is unoccupied (step 1 in Figure 5), as in the apo-EcTH state, dIII remains attached to the membrane domain dII near the cytoplasmic entrance of the proton channel. This attachment closes the proton channel on the cytoplasmic side while keeping it open to the periplasm, allowing His91 in the proton channel to equilibrate with the external (periplasmic) proton environment. The proton-motive force under these conditions favors protonation of His91.
(3) A second consequence of dIII remaining attached to dII is that the NADP(H)-binding site is in rapid equilibrium with cytoplasmic NADP^+^ and NADPH. Under conditions where [NADP^+^] >> [NADPH], NADP^+^ binding is favored (steps 1→2 in Figure 5).
(4) The combination of His91 protonation and NADP^+^ binding to dIII triggers its detachment from the membrane domain dII (steps 2→3 in Figure 5). Although dIII can adopt multiple positions and orientations, the (dI)_2_ dimer must shift to allow dIII to disengage from dII. A key outcome of this detachment is the opening of the proton channel on the cytoplasmic side and its closure on the periplasmic side. His91 now equilibrates with the cytoplasm, and the proton-motive force drives proton release into the cytoplasm.
(5) A second consequence of dIII detachment from dII is that the NADP(H)-binding site becomes occluded and no longer exchanges with cytoplasmic NADP^+^ or NADPH on the timescale of catalytic turnover (steps 3, 4, 5, and 6 in Figure 5).
(6) Once detached, dIII and the (dI)_2_ dimer can transiently form a complex, positioning the bound NADH in (dI)_2_ near the NADP^+^ in dIII to enable rapid hydride transfer (steps 3→4 and 5→6 in Figure 5). The NAD^+^ product formed in (dI)_2_ rapidly dissociates and, under the specified conditions, is replaced by NADH, whereas the NADPH product formed in dIII remains trapped in the occluded site (steps 4→5 and 6→7 in Figure 5).
(7) Because the (dI)_2_ dimer cannot simultaneously engage both copies of dIII, the two detached dIII domains interact with (dI)_2_ sequentially. Following these interactions, both copies retain occluded NADPH (step 6 in Figure 5).
(8) With NADPH bound in dIII and His91 deprotonated, dIII reattaches to dII (6→7 in Figure 5). Reattachment closes the proton channel on the cytoplasmic side, opens it to the periplasm, and enables rapid protonation of His91 from the periplasm, an event favored by the proton-motive force (steps 7→1 in Figure 5).
(9) Reattachment of dIII to dII also restores rapid equilibration of the NADP(H)-binding site with cytoplasmic NADP^+^/NADPH (steps 7→1→2 in Figure 5). Under the specified conditions, NADPH dissociates and is replaced by NADP^+^, initiating another round of catalysis.

### Limitations of the study

Several limitations should be noted. First, all structures were determined at pH 8.0, and the protonation state of His91 is not known. Second, although the major ligand-dependent conformational transitions observed in the chimeric construct were also detected in the wild-type enzyme, the artificial linker may constrain or bias the relative movements of dIII and dI, and the chimera retains only approximately one-third of wild-type activity. Third, the (dI)_2_ density in several wild-type datasets was weaker than that of dII and dIII, limiting modeling precision. Finally, all structures are static snapshots captured in detergent solution in the absence of a transmembrane proton gradient. Further studies, for example using proteoliposome reconstitution under a controlled proton-motive force, would be helpful to assess the coupling mechanism under more physiological conditions.

## Resource availability

### Lead contact

Requests for further information and resources should be directed to and will be fulfilled by the lead contact, Jiapeng Zhu (zhujiapeng@fy.ahmu.edu.cn).

### Materials availability

This study did not generate new unique reagents.

### Data and code availability

• The atomic coordinates for EcTH have been deposited in the Protein DataBank and are available under the following accession codes: 9UUY (WT apo-EcTH major), 9UV7 (Single-subunit apo-EcTH minor), 9UV2 (Single-subunit apo-EcTH major), 9WWS (WT EcTH double dIIIs attached to dII in the presence of both NADP^+^ and NAD^+^), 9WWU (WT EcTH single dIII attached to dII in the presence of both NADP^+^ and NAD^+^), 9WWV (WT EcTH the dissociated (dI)_2_ in the presence of both NADP^+^ and NAD^+^), 9UV3 (Single-subunit EcTH in the presence of palmitoyl-CoA), 9UVB (EcTH the dissociated (dI)_2_ in the presence of both NADPH and NADP^+^), and 9WWW (EcTH double dIIIs attached to dIIs state in the presence of NADPH).
• The corresponding cryo-EM maps were deposited in the Electron Microscopy DataBank (EMDB) with the following accession numbers: EMD-64520 (WT apo-EcTH major), EMD-64527 (Single-subunit apo-EcTH minor), EMD-64521 (Single-subunit apo-EcTH major), EMD-66319 (WT EcTH double dIIIs attached to dII in the presence of both NADP^+^ and NAD^+^), EMD-66321 (WT EcTH single dIII attached to dII in the presence of both NADP^+^ and NAD^+^), EMD-66322 (WT EcTH the dissociated (dI)_2_ in the presence of both NADP^+^ and NAD^+^), EMD-64522 (Single-subunit EcTH in the presence of palmitoyl-CoA), EMD-64533 (EcTH single dIII attached to dII state in the presence of both NADPH and NADP^+^), EMD-64534 (EcTH double dIIIs detached to dIIs state in the presence of both NADPH and NADP^+^), EMD-64530 (EcTH the dissociated (dI)_2_ in the presence of both NADPH and NADP^+^), EMD-64531 (EcTH single dIII attached to dII state in the presence of NADPH), and EMD-66323 (EcTH double dIIIs attached to dIIs state in the presence of NADPH).
• This paper does not report original code.
• Any additional information required to reanalyze the data reported in this paper is available from the lead contact upon request.

## Acknowledgments

We thank Dr. Yongxiang Gao at the Cryo-EM Center at the University of Science and Technology of China (USTC) and Dr. Shenping Wu at the Yale West Campus Advanced Biosciences Center for their assistance with the experiments. This work was supported by the 10.13039/501100001809National Natural Science Foundation of China (grant nos. 32671722 and 32201038 to J. L.); Start-up funds from the First Affiliated Hospital of 10.13039/501100002947Anhui Medical University (to J.Z.); National Science Foundation grant 1616874 (to R.B.G.); and Start-up funds from the University of Science and Technology of China (to K.Z.).

## Author contributions

J.Z., R.B.G., and K.Z. designed the project. J.L. prepared all EM samples, processed the EM data, and built the models, assisted by J.Z. S.H. generated DNA constructs and conducted biochemical characterization, assisted by R.H. All authors participated in data interpretation. J.L., J.Z., and R.B.G. prepared the manuscript, assisted by other co-authors.

## Declaration of interests

The authors declare no competing interests.

## STAR★Methods

### Key resources table

**Table:** 

REAGENT or RESOURCE	SOURCE	IDENTIFIER
**Chemicals, peptides, and recombinant proteins**		

Isopropyl-β-D-thiogalactopyranoside (IPTG)	Solarbio	Cat#I8070
Protease Inhibitor Cocktail	Sigma-Aldrich	Cat#4693132001
LMNG (Lauryl Maltose Neopentyl Glycol)	Anatrace	Cat#NG310
3-Acetylpyridine adenine dinucleotide (APAD^+^)	Sigma-Aldrich	Cat#A5251
NADPH	APExBIO Technology LLC	Cat#C3974
NADP^+^	Sigma-Aldrich	Cat#N5755
NAD^+^	Sigma-Aldrich	Cat#NAD100-RO
Palmitoyl-CoA	Sigma-Aldrich	Cat#870716P
Asolectin	Sigma-Aldrich	Cat#P5638
Imidazole	Sigma-Aldrich	Cat#I202
Ampicillin sodium	Sangon Biotech	Cat#A600064
Chloramphenicol	Sangon Biotech	Cat#A429048

**Critical commercial assays**		

Ni-NTA Agarose resin	Qiagen	Cat#30230
100 kDa MWCO ultrafiltration concentrator (e.g., Amicon Ultra)	Millipore	Cat#UFC910024
Quantifoil R 2/1 gold cryo-EM grids, 300-mesh	Quantifoil Micro Tools GmbH	Cat#X-102-Au300

**Deposited data**		

Atomic coordinates for EcTH (multiple cryo-EM conformations)	This paper	PDB: 9UUY; 9UV7; 9UV2; 9WWS; 9WWU; 9WWV; 9UV3; 9UVB; 9WWW
Cryo-EM density maps for EcTH	This paper	EMD-64520; EMD-64527; EMD-64521; EMD-66319; EMD-66321; EMD-66322; EMD-64522; EMD-64533; EMD-64534; EMD-64530; EMD-64531; EMD-66323

**Experimental models: Organisms/strains**		

*E. coli* Strain THO: C43(DE3) Δ*cyo* Δ*pntAB* (chloramphenicol resistant)	This paper	N/A

**Oligonucleotides**		

Primers for pET16b-EcTH and pET16b-single-subunit-EcTH construction, see Table S2	This paper	N/A

**Recombinant DNA**		

Plasmid: pSA2 (carrying *pntAB* operon with His-tag)	Lab gift, Egorov et al.^38^	N/A
Plasmid pET16b-EcTH (WT *pntAB* inserted into pET16b)	This paper	N/A
Plasmid pET16b-single-subunit-EcTH (His-tag-*pntB*-linker-*pntA*)	This paper	N/A

**Software and algorithms**		

SerialEM	Mastronarde^39^	https://bio3d.colorado.edu/SerialEM
MotionCor2	Zheng et al.^40^	https://emcore.ucsf.edu/ucsf-motioncor2/
Gctf	Zhang^41^	https://www.mrc-lmb.cam.ac.uk/kzhang/Gctf/
Gautomatch	Zhang Lab	https://www.mrc-lmb.cam.ac.uk/kzhang/
RELION	Scheres^42^	https://relion.readthedocs.io
cryoSPARC	Punjani et al.^43^	https://cryosparc.com
UCSF Chimera	Pettersen et al.^44^	https://www.cgl.ucsf.edu/chimera/
AlphaFold	Jumper et al.^45^	https://alphafold.ebi.ac.uk
Coot	Emsley et al.^34^	https://www2.mrc-lmb.cam.ac.uk/personal/pemsley/coot/
Phenix.real_space_refine	Adams et al. (Phenix Software Suite)	https://phenix-online.org
MOLEonline	Pravda et al.^46^	https://mole.upol.cz
UCSF ChimeraX	Goddard et al.^47^	https://www.cgl.ucsf.edu/chimerax/
PyMOL	Schrödinger, LLC	https://pymol.org
Movavi Video Editor	Movavi Software Inc.	https://www.movavi.com

**Other**		

ENrich™ SEC 650 10 × 300 Column	Bio-Rad	Cat#7801650
FEI Vitrobot Mark IV	Thermo Fisher Scientific	N/A
Titan Krios transmission electron microscope	Thermo Fisher Scientific	N/A
Gatan K3 direct electron detector	Gatan	N/A
Microfluidizer cell homogenizer	Microfluidics	N/A
Agilent 8453 UV-Visible Spectrophotometer	Agilent Technologies	N/A

### Experimental model and study participant details

#### Bacterial strain and growth conditions

The *E. coli* transhydrogenase *pnt* genes were subcloned from pSA2 carrying *pntAB* operon with a His-tag into pET16b using the *Nco*I and *Bam*HI sites to generate pET16b-EcTH.^38^ The *pntAB* knockout strain (THO, C43(DE3) Δ*cyo* Δ*pntAB*), in which the *pnt* genes were replaced by a chloramphenicol resistance gene, was transformed with the pET16b-EcTH plasmid encoding WT *E. coli* transhydrogenase.^48^ Transformed cells were plated onto LB agar containing 30 μg/ml chloramphenicol and 100 μg/ml ampicillin (chl^30^/amp^100^) and incubated at 37 °C overnight. A single colony was inoculated into 5 ml LB (chl^30^/amp^100^) and incubated at 37 °C overnight. The overnight culture was expanded to 50 ml LB (chl^30^/amp^100^) and incubated again overnight. The following morning, 1 ml of the culture was used to inoculate 100 ml LB (chl^30^/amp^100^) and grown for 3 h. These precultures were then used to inoculate expression cultures (10 ml per liter of culture volume), which were incubated at 37 °C. When the OD_600_ reached 0.6-0.8, protein expression was induced by adding 0.2 mM isopropyl-β-D-thiogalactopyranoside (IPTG), followed by overnight incubation at 23 °C.

To generate a single-subunit EcTH mimicking the protozoan transhydrogenase, the *pntB* and the *pntA* genes were fused using a linker sequence derived from the protozoan *Paratrimastix pyriformis* transhydrogenase (TQGGAAPAPAAAAATERTPLVQDAPKPEVQETYPEPK). A His-tag was added to the N-terminus of the fused *pntB-*linker*-pntA* construct. The resulting fusion construct was cloned into the pET16b vector using the *Nco*I and *Bam*HI restriction sites, yielding the expression plasmid pET16b-single-subunit-EcTH. This plasmid was subsequently transformed into the *E. coli* THO strain. Transformed cells were cultured under the same conditions used for WT EcTH expression.

### Method details

#### Membrane preparation and protein purification

Cells were grown overnight and harvested by centrifugation at 5,000 × g for 12 min at 4 °C. The harvested cells were resuspended in lysis buffer containing 50 mM KH_2_PO_4_ (pH 8.0), 1 mM EDTA, and 1% Protease Inhibitor Cocktail (Sigma). This suspension was passed five times through a microfluidizer at 15,000 psi to disrupt the cells. The lysate was centrifuged at 20,000 × g for 20 min to remove cell debris, and membranes were collected by ultracentrifugation at 180,000 × g for 4 h and stored at -80°C. On the day of purification, the membrane pellet was resuspended in 50 mM potassium phosphate buffer (pH 8.0) and solubilized with 2% LMNG on ice for 1 h. Insoluble material was removed by ultracentrifugation at 180,000 × g for 20 min. The solubilized fraction was loaded onto a Ni-nitrilotriacetic acid (Ni-NTA) affinity column (Qiagen) pre-equilibrated with 50 mM potassium phosphate buffer (pH 8.0) containing 0.1% LMNG. Bound proteins were eluted with 250 mM imidazole in the same buffer. Eluted peak fractions were pooled, concentrated, and further purified by size-exclusion chromatography on an ENrich^TM^ SEC 650 10 × 300 Column (Bio-Rad, Cat# 7801650) equilibrated with gel filtration buffer containing 20 mM Tris-HCl (pH 7.5), 50 mM NaCl, and 0.03% LMNG. The most concentrated and purest fractions were pooled and concentrated to 2 mg/ml using a 100 kDa cut-off concentrator (Millipore) and used immediately for cryo-EM grid preparation.

#### Reverse transhydrogenase activity

Reverse transhydrogenase activity was measured spectrophotometrically by monitoring the reduction of 3-acetylpyridine adenine dinucleotide (APAD^+^) by NADPH at 375 nm and 37 °C, using an Agilent 8453 UV-Visible spectrophotometer and an extinction coefficient of 6.07 mM^-1^cm^-1^. The reaction mixture contained 25 mM HEPES (pH 7.5), 50 mM KCl, 125 μg/ml asolectin, 0.1% LMNG, 100 μM NADPH, and 100 μM APAD^+^.

#### Cryo-EM grid preparation and data collection

Stock solutions of NADPH and NADP^+^ were mixed with purified WT and single-subunit EcTH protein solutions, respectively, to yield final mixtures containing ∼2 mg/ml transhydrogenase, 3 mM NADPH and 3 mM NADP^+^. For the NADPH-only condition, stock NADPH was mixed with the single-subunit transhydrogenase solution to a final concentration of ∼2 mg/ml protein and 3 mM NADPH. For inhibitor-bound conditions, stock palmitoyl-CoA was mixed with the single-subunit transhydrogenase solution to a final concentration of ∼2 mg/ml protein and 1 mM palmitoyl-CoA. The mixtures were incubated on ice for ∼15 min, except those containing palmitoyl-CoA, which were incubated for 1 h prior to grid preparation. For each sample, 3 μl was applied to a freshly glow-discharged 300-mesh Quantifoil R 2/1 gold grid and blotted for 2.5–4 s with a blotting force of −4 at 8°C and 100% humidity in a FEI Vitrobot Mark IV (Thermo Fisher). The apo protein sample was prepared using identical conditions. Grids were flash-frozen in liquid ethane and stored in liquid nitrogen until data collection. Cryo-EM data were collected automatically using a Titan Krios transmission electron microscope (Thermo Fisher) equipped with a Gatan K3 direct electron detector, operated in counting mode. Automated data acquisition was performed using SerialEM software.^39^ Detailed data collection parameters are provided in Table S1.

#### Data processing

Beam-induced motion in each micrograph was corrected using MotionCor2,^40^ and contrast transfer function (CTF) parameters were estimated using Gctf.^41^ Initially, 384,565 particles were picked from 1,000 micrographs using autopicking and initial 2D class averages were generated in RELION^42^ to serve as templates for automated particle picking across the full dataset using Gautomatch (https://www.mrc-lmb.cam.ac.uk/kzhang/). A total of 6,079,195 particles were picked from the WT apo-EcTH dataset, 5,262,868 particles from the single-subunit apo-EcTH dataset, 3,109,377 particles from the WT EcTH _NADP+/NAD+_ dataset, 6,939,285 particles from the single-subunit EcTH_NADPH/NADP+_ dataset, 6,592,383 particles from the WT EcTH_NADPH/NADP+_ dataset, 8,668,929 particles from the single-subunit EcTH_NADPH_ dataset and 2,585,181 particles from the single-subunit EcTH_palmitoyl-CoA_ dataset.

Subsequent data processing was performed using cryoSPARC,^43^ including particle extraction, 2D classification, *ab initio* reconstruction, heterogeneous refinement, local refinement and per-particle CTF refinement. For the WT apo-EcTH dataset, 204,263 particles were used to reconstruct a 3.85 Å resolution map corresponding to the double dIIIs face-down and the (dI)_2_-detached WT apo-EcTH (major) conformation (Figure S1). For the single-subunit apo-EcTH dataset, two final classes of 42,826 and 164,138 particles were used to reconstruct two high-resolution 3D maps: a 3.53 Å resolution map of the entire single-subunit EcTH (minor) adopting the double dIIIs face-down conformation and a 2.86 Å resolution map of the (dI)_2_-detached single-subunit apo-EcTH (major), respectively (Figure S2). For the WT EcTH NADP^+^/ NAD^+^ dataset, four final classes were used to reconstruct a 2.84 Å resolution map of ${\text{WT}\;\text{EcTH}}_{{{\text{NADP}}^{+}/\text{NAD}}^{+}}^{\text{double}\;\text{dIIIs}\;\text{attached}}$, a 3.3 Å resolution map of ${\text{WT}\;\text{EcTH}}_{{{\text{NADP}}^{+}/\text{NAD}}^{+}}^{\text{single}\;\text{dIII}\;\text{attached}}$, a 3.38 Å resolution map of ${\text{WT}\;\text{EcTH}}_{{{\text{NADP}}^{+}/\text{NAD}}^{+}}^{\text{detached}{\;\left(\text{dI}\right)}_{2}}$, and a 4.91 Å resolution map of ${\text{WT}\;\text{EcTH}}_{{{\text{NADP}}^{+}/\text{NAD}}^{+}}^{\text{minor}}$, respectively (Figure S3). For the WT EcTH NADPH/NADP^+^ dataset and the single-subunit EcTH NADPH/NADP^+^ dataset, they were processed separately (Figure S4), revealing four primary conformations including but not limited to: single dIII attached to dII (${\text{EcTH}}_{{\text{NADPH}/\text{NADP}}^{+}}^{\text{single}\;\text{dIII}\;\text{attached}}$), double dIIIs detached from dII (${\text{EcTH}}_{{\text{NADPH}/\text{NADP}}^{+}}^{\text{double}\;\text{dIIIs}\;\text{detached}}$), (dI)_2_ detached state and dIII detached state. The conformation identical to the intact apo-EcTH minor form is not shown in Figure S4E. For the single-subunit EcTH NADPH dataset, three final classes were used to reconstruct a 3.55 Å resolution map of single-subunit ${\text{EcTH}}_{\text{NADPH}}^{\text{single}\;\text{dIII}\;\text{attached}}$, a 3.84 Å resolution map of single-subunit ${\text{EcTH}}_{\text{NADPH}}^{\text{double}\;\text{dIIIs}\;\text{attached}}$ and a 7.58 Å resolution map of single-subunit ${\text{EcTH}}_{\text{NADPH}}^{\text{minor}}$, respectively (Figure S5). For the single-subunit EcTH palmitoyl-CoA dataset, two final classes were used to reconstruct a 2.97 Å resolution map of single-subunit ${\text{EcTH}}_{\text{palmitoyl}-\text{CoA}}^{\text{major}}$ and a 3.69 Å resolution map of single-subunit ${\text{EcTH}}_{\text{palmitoyl}-\text{CoA}}^{\text{minor}}$, respectively (Figure S7).

#### Model building, refinement, and validation

An AlphaFold^45^-predicted model of each subunit was initially fitted into the corresponding cryo-EM density map as a rigid body using UCSF Chimera.^44^ Side chains were manually adjusted and refined to correct local errors and ensure optimal fit to the density using Coot.^34^ The inhibitor palmitoyl-CoA ligands were manually modeled and incorporated into the structures. The complete models were then refined in real space using Phenix.real_space_refine, applying appropriate geometric restraints.

#### Structure analysis and preparation of figures

The proton translocation pore was predicted and analysed using a MOLEonline server.^46^ Models and density maps were visualized using UCSF ChimeraX^47^ and PyMOL (The PyMOL Molecular Graphics System, Schrödinger, LLC) for analysis, figure preparation. Videos were generated using UCSF ChimeraX and edited with Movavi Video Editor (26.17.0).

### Quantification and statistical analysis

For cryo-EM data processing, resolution estimates were based on the gold-standard Fourier shell correlation (FSC) criterion at 0.143. All data processing and model refinement statistics are summarized in Table S1.

## Supplemental information

Document S1. Figures S1–S13

Table S1. Cryo-EM data collection, refinement, and validation statistics

Table S2. Oligonucleotides used in this study
