# Supplementary material for: Structures of multiple states of the nicotinamide nucleotide transhydrogenase from Escherichia coli
Source: iScience. 2026 Sep 24;29(10):117657. doi: 10.1016/j.isci.2026.117657 (PMC13635403; doi:10.1016/j.isci.2026.117657)
Supplement: Supplementary file 1 [file mmc1.pdf]

iScience, Volume *29*

Supplemental information

Structures of multiple states of the nicotinamide nucleotide transhydrogenase from *Escherichia coli*

Jiao Li, Sangjin Hong, Rui Hao, Kai Zhang, Robert B. Gennis, and Jiapeng Zhu

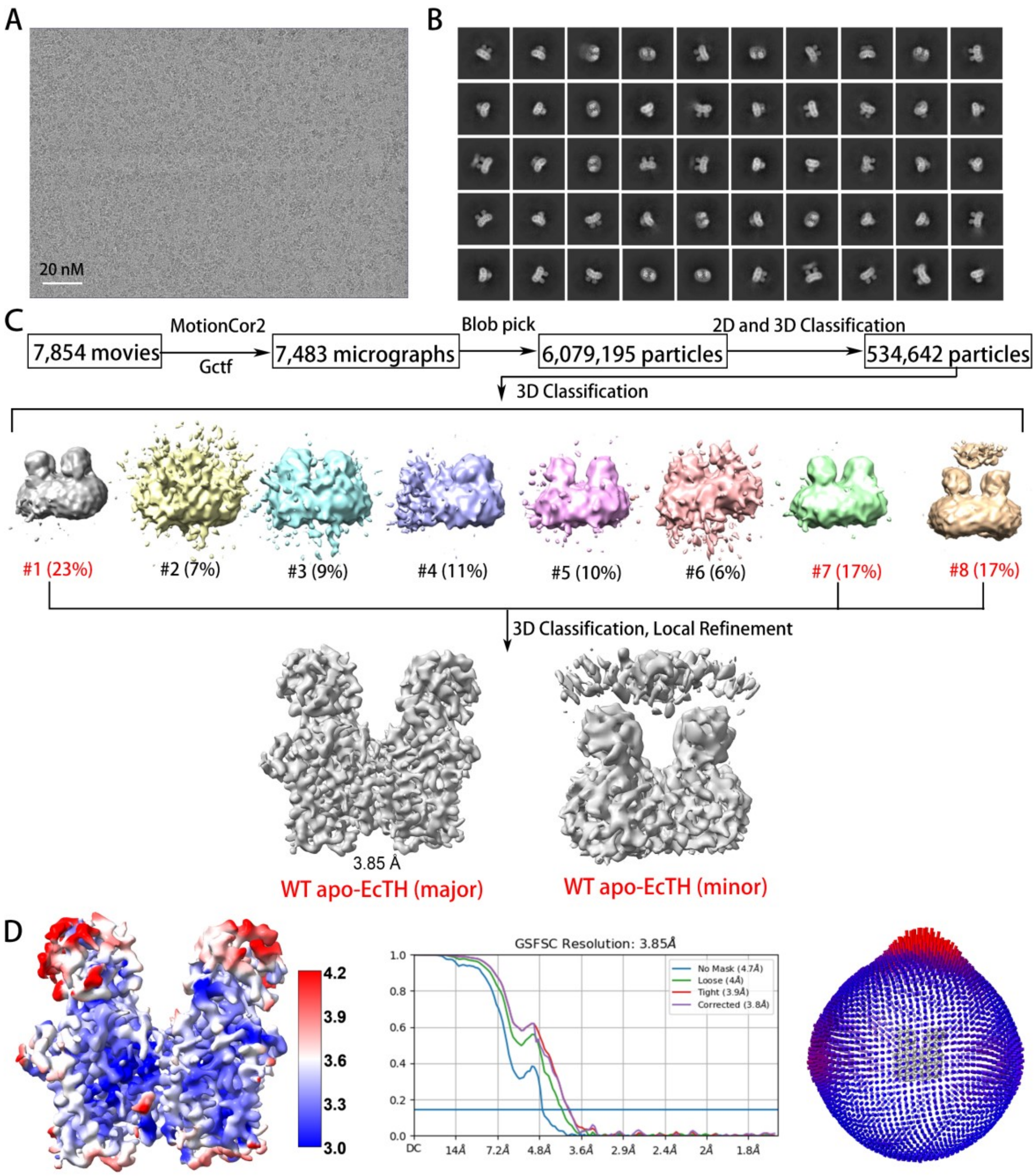

Fig. S1. Classification and refinement of the cryo-EM maps for WT apo-EcTH. (A) Representative micrograph of WT apo-EcTH. The scale bar represents 20 nm. (B) Class averages after the final round of two-dimensional classification, sorted in descending order by the number of particles in each class. (C) Workflow implemented in cryoSPARC v4.5.3 to obtain maps of the $(dI)_2$-detached WT apo-EcTH (major) and the entire WT apo-EcTH (minor)，respectively. (D) Local resolution map, gold standard Fourier shell correlation (FSC) curve calculated in cryoSPARC, and orientation plot of particles included in the final 3D reconstruction for the WT apo-EcTH (major).

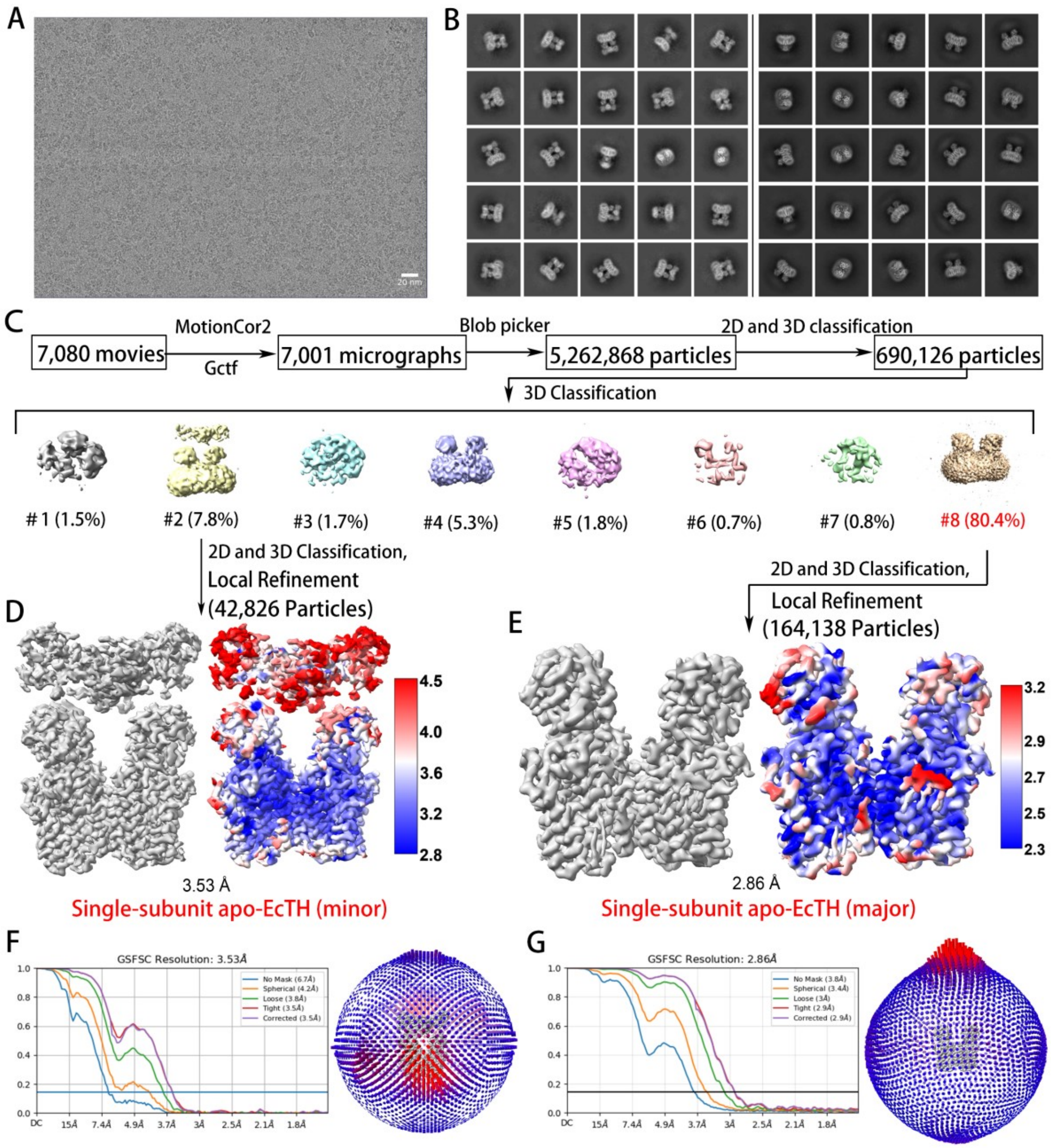

Fig. S2. Classification and refinement of the cryo-EM maps for single-subunit apo-EcTH. (A) Representative micrograph of the single-subunit apo-EcTH. The scale bar represents 20 nm. (B) Class averages after the final round of two-dimensional classification for two different conformations, sorted in descending order by the number of particles in each class. (C) Workflow implemented in cryoSPARC v4.5.3 to obtain maps of the entire single-subunit apo-EcTH (minor) (D) and the $(dI)_2$-detached single-subunit apo-EcTH (major) (E). (F and G) Gold-standard FSC curves from cryoSPARC and orientation plots of particles used for the final 3D reconstruction of the entire single-subunit apo-EcTH minor (F) and the $(dI)_2$-detached single-subunit apo-EcTH major (G), respectively.

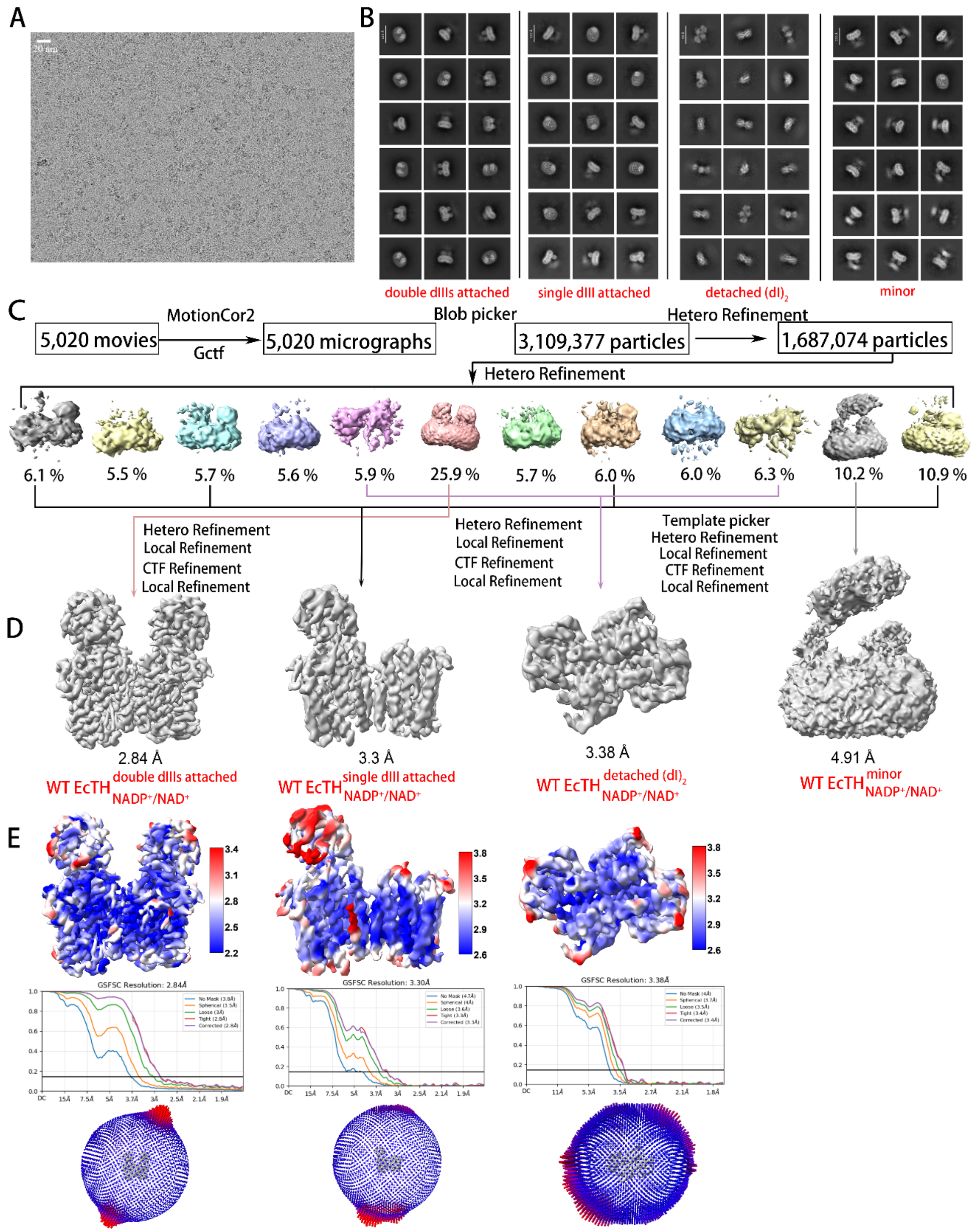

Fig. S3. Classification and refinement of the cryo-EM maps for WT EcTH in the presence of both NADP$^+$ and NAD$^+$. (A) Representative micrograph of WT EcTH in the presence of both NADP$^+$ and NAD$^+$. The scale bar represents 20 nm. (B) Class averages after the final round of two-dimensional classification for different conformations, sorted in descending order by the number of particles in each class. (C and D) Workflows implemented in cryoSPARC v4.5.3 to obtain four conformational maps of WT EcTH in

the presence of both $NADP^+$ and NAD^+. (E) Local resolution maps of the three high-resolution conformations of WT EcTH in the presence of both $NADP^+$ and NAD^+, along with the corresponding gold-standard FSC curves from cryoSPARC and orientation plots of particles included in the final 3D reconstruction.

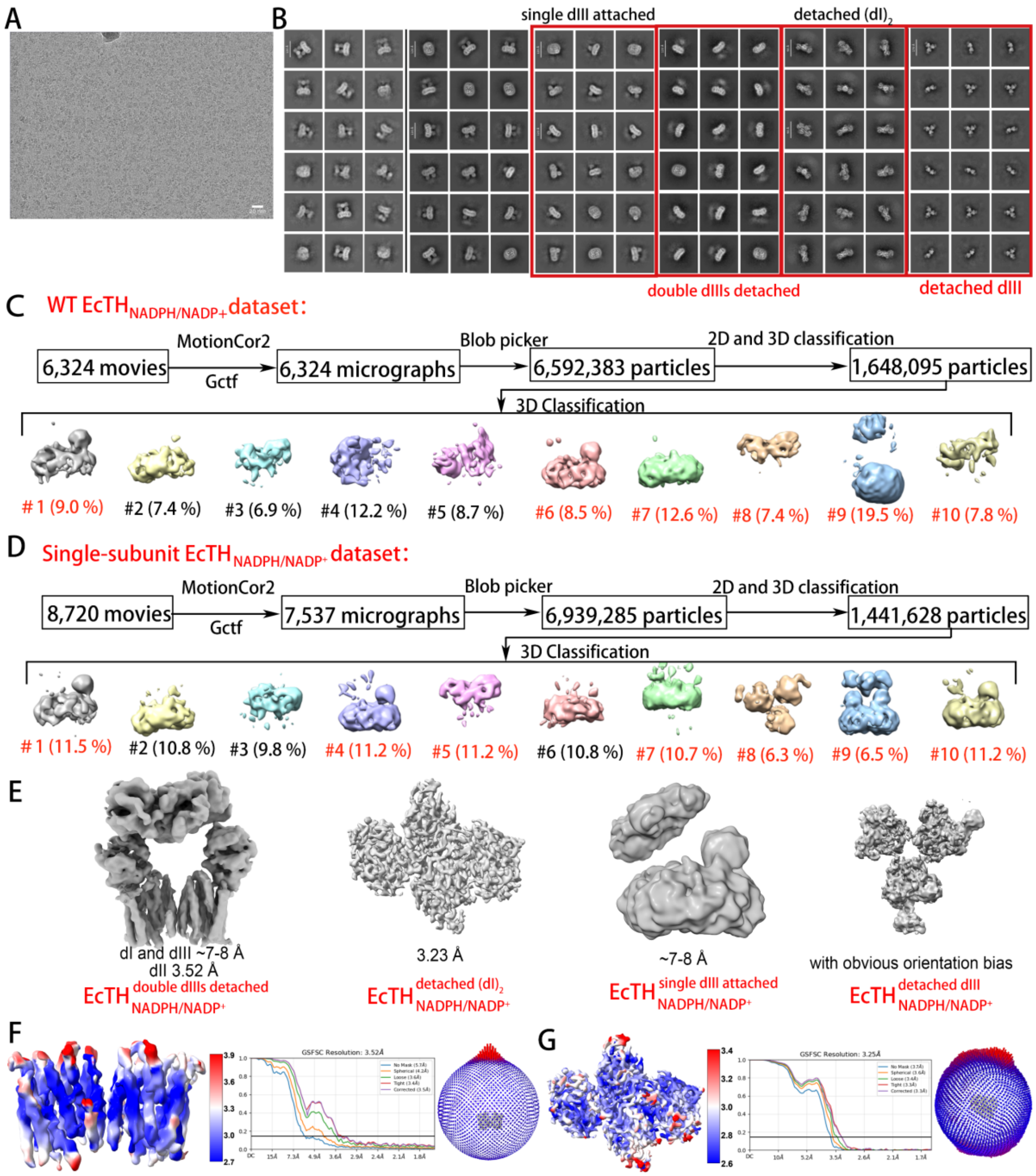

Fig. S4. Classification and refinement of the cryo-EM maps for WT and single-subunit EcTH in the presence of both NADPH and NADP$^+$. (A) Representative micrograph of single-subunit EcTH in the presence of both NADPH and NADP$^+$. The scale bar represents 20 nm. (B) Class averages after the final round of two-dimensional classification for different conformations, sorted in descending order by the number of particles in each class. (C and D) Workflows implemented in cryoSPARC v4.5.3 to obtain maps of WT EcTH (C) and single-subunit EcTH (D) in the presence of both NADPH and NADP$^+$. (E) Four primary conformational maps of the single-subunit EcTH in the presence of both NADPH and NADP$^+$. (F and G) Local resolution maps, gold-standard FSC curves

from cryoSPARC, and orientation plots of particles included in the final 3D reconstruction for the high-resolution dII domains of the $EcTH^{\text{double dIIIs detached}}_{\text{NADPH/NADP}^+}$ (F) and $EcTH^{\text{double dIIIs detached}}_{\text{NADPH/NADP}^+}$ (G), respectively.

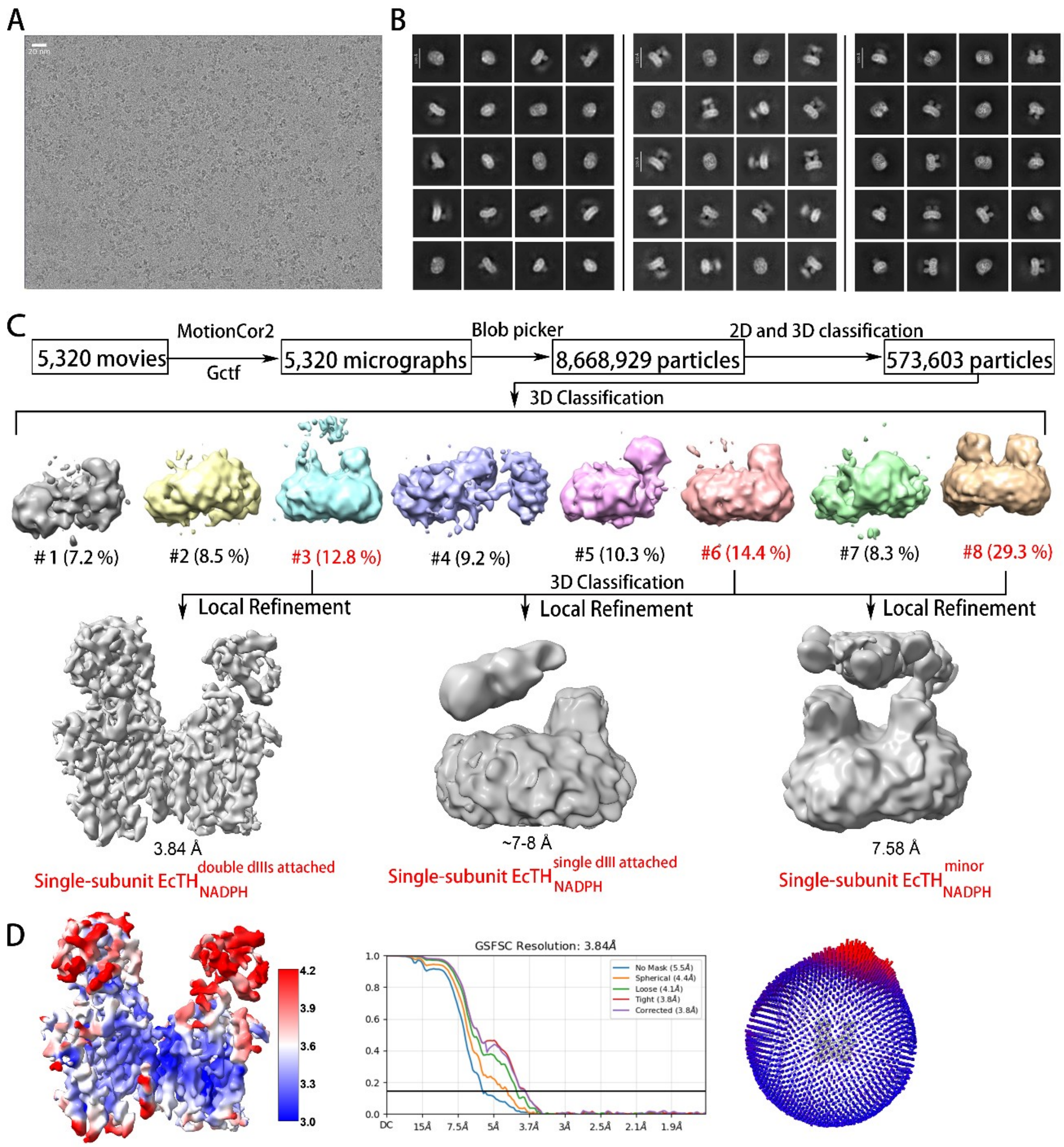

Fig. S5. Classification and refinement of the cryo-EM maps for single-subunit EcTH in the presence of NADPH. (A) Representative micrograph of the single-subunit EcTH in the presence of NADPH. The scale bar represents 20 nm. (B) Class averages after the final round of two-dimensional classification for different conformations, sorted in descending order by the number of particles in each class. (C) Workflow implemented in cryoSPARC v4.5.3 to obtain maps of single-subunit EcTH in the presence of NADPH. (D) Local resolution map, gold-standard FSC curve from cryoSPARC, and orientation plot of particles included in the final 3D reconstruction are provided for the high-resolution $\mathrm{EcTH}_{\mathrm{NADPH}}^{\mathrm{double\ dIIIs\ attached}}$, respectivley.

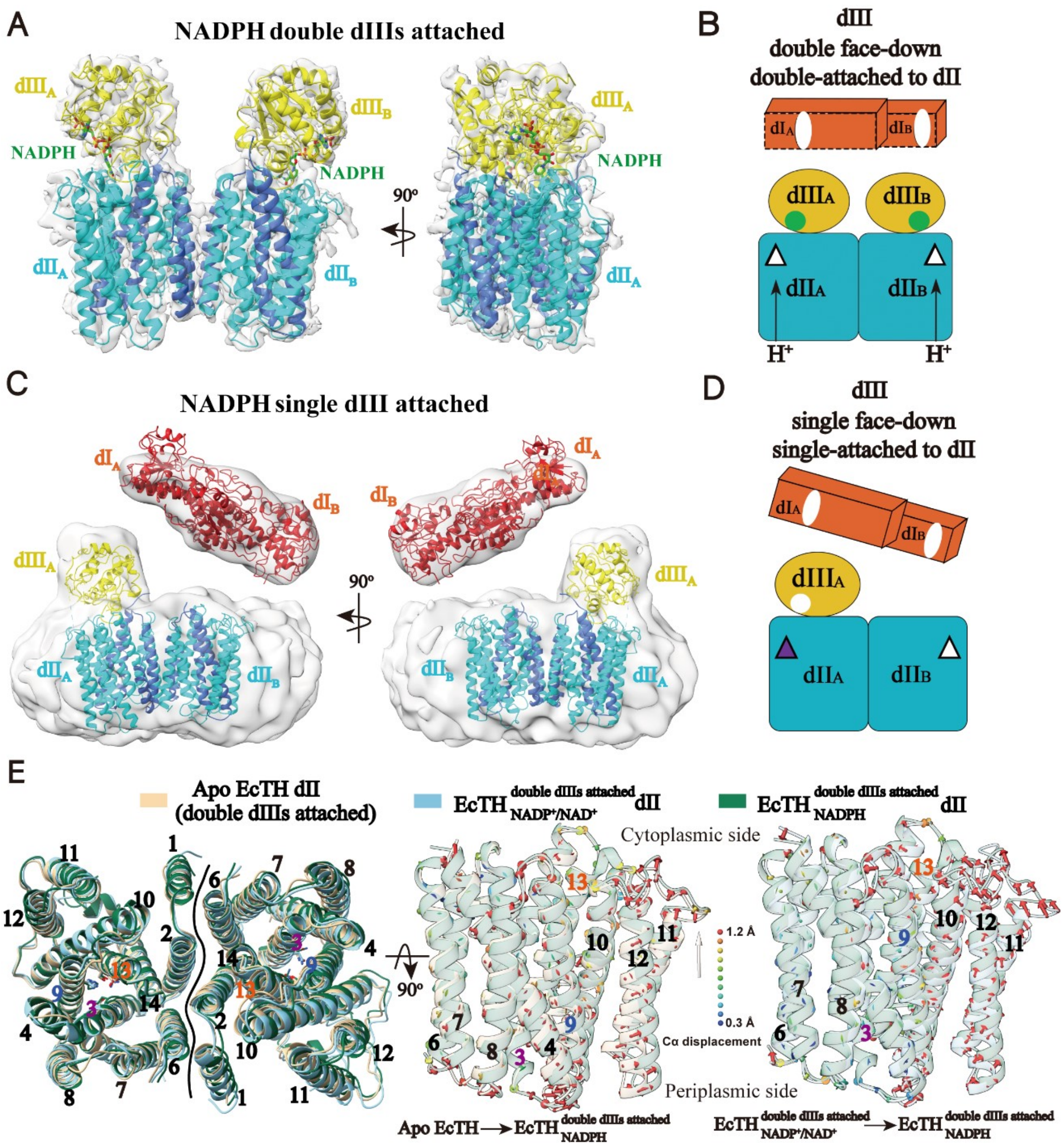

Fig. S6. Conformational states of single-subunit EcTH in the presence of NADPH. (A) Structure of single-subunit $EcTH_{NADPH}^{double\ dIIIs\ attached}$. Both dIII copies adopt a face-down orientation and attach to dII, with each dIII binding one NADPH molecule in its NADP(H)-binding site. The $(dI)_2$ dimer exhibits dynamic characteristics with weak electron density (Fig. S6B), precluding determination of an intact structure containing the $(dI)_2$ dimer. (B) Schematic representation of single-subunit $EcTH_{NADPH}^{double\ dIIIs\ attached}$. The green circle in dIII represents NADPH-bound state and the white triangle denotes deprotonated His91 in the proton channel. (C) Structure of single-subunit $EcTH_{NADPH}^{single\ dIII\ attached}$. One copy of dIII is completely detached from dII, whereas the other copy remains attached in a face-down orientation. The $(dI)_2$ dimer tilts toward the side where dIII is detached. (D)

Schematic representation of single-subunit $\mathrm{EcTH}_{\mathrm{NADPH}}^{\text{single dIII attached}}$. Purple and white triangles represent protonated and deprotonated His91, respectively, in the proton channel. (E) Superposition of the dII domains from $\mathrm{EcTH}_{\mathrm{NADP^+/NAD^+}}^{\text{double dIIIs attached}}$ and $\mathrm{EcTH}_{\mathrm{NADPH}}^{\text{double dIIIs attached}}$ onto the corresponding domain in the single-subunit apo-EcTH, viewed from the periplasmic side (left panel). All structures were aligned using the left-side proton channel helices TM3, TM9, and TM13. The conformational changes of the dII on the right side of the left panel are shown separately: transitions from the apo state to the $\mathrm{EcTH}_{\mathrm{NADPH}}^{\text{double dIIIs attached}}$ state (middle panel) and from the $\mathrm{EcTH}_{\mathrm{NADP^+/NAD^+}}^{\text{double dIIIs attached}}$ state to the $\mathrm{EcTH}_{\mathrm{NADPH}}^{\text{double dIIIs attached}}$ state (right panel). Displacement magnitudes are colored as in Fig. 2E.

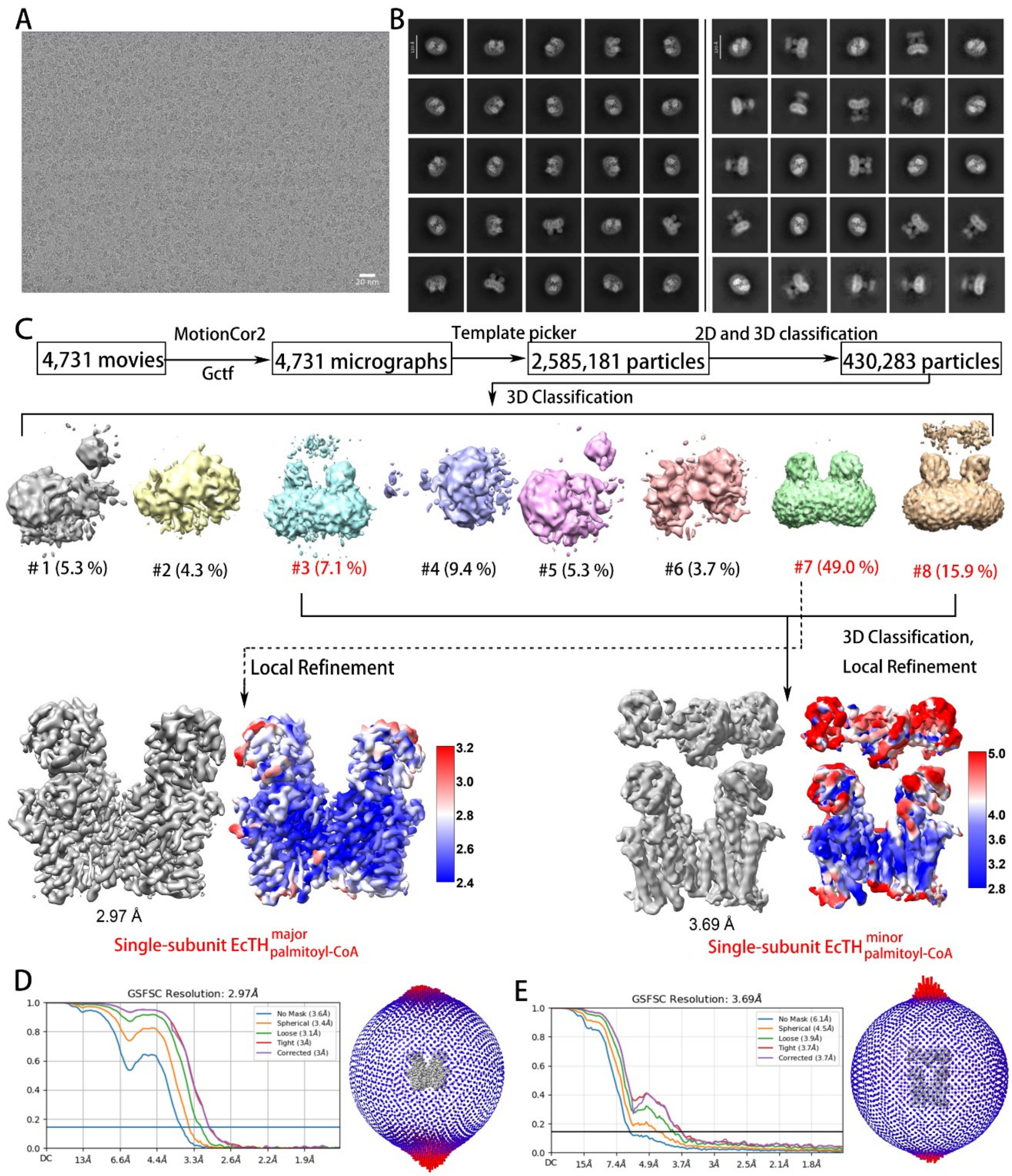

Fig. S7. Classification and refinement of the cryo-EM maps for single-subunit EcTH in the presence of palmitoyl-CoA. (A) Representative micrograph of the single-subunit EcTH in the presence of palmitoyl-CoA. The scale bar represents 20 nm. (B) Class averages after the final round of two-dimensional classification for different conformations, sorted in descending order by the number of particles in each class. (C) Workflow implemented in cryoSPARC v4.5.3 to obtain maps of single-subunit EcTH in the presence of palmitoyl-CoA. (D and E) Gold-standard FSC curves from cryoSPARC and orientation plots of particles used for the final 3D reconstruction of the major (D) and the minor (E) classes of single-subunit $EcTH_{palmitoyl\text{-}CoA}$, respectively.

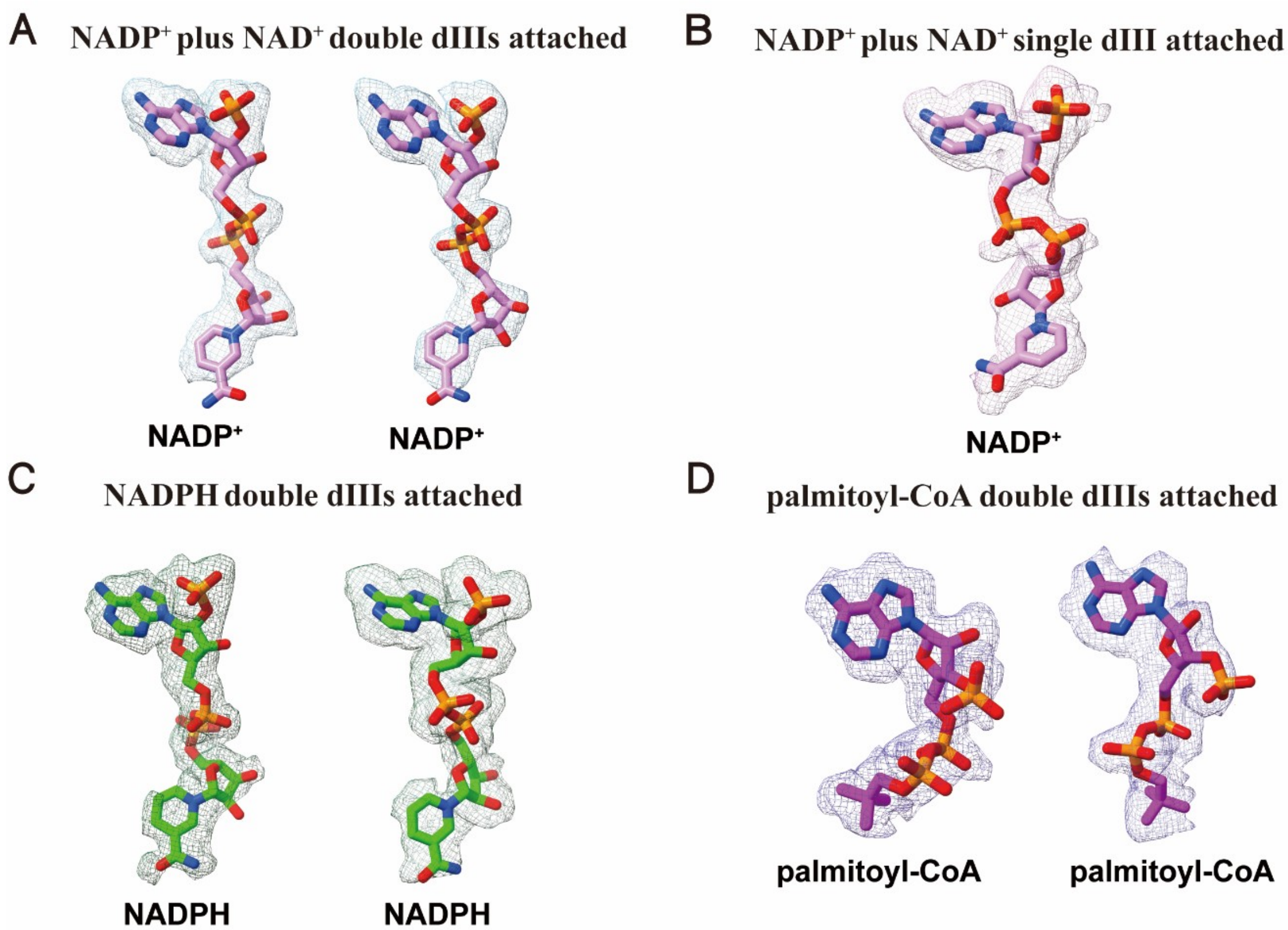

Fig. S8. Cryo-EM density maps of substrates in dIII in different conformational states. Cryo-EM density maps are shown for (A) each $NADP^+$ bound to the two domains of double attached dIIIs in WT $EcTH_{NADP^+/NAD^+}^{double\ dIIIs\ attached}$, (B) one $NADP^+$ bound to single attached dIII in WT $EcTH_{NADP^+/NAD^+}^{single\ dIII\ attached}$, (C) each NADPH bound to the two domains of double attached dIIIs in single-subunit $EcTH_{NADPH}^{double\ dIIIs\ attached}$, and (D) each palmitoyl-CoA bound to the two domains of double attached dIIIs in single-subunit $EcTH_{palmitoyl-CoA}^{major}$.

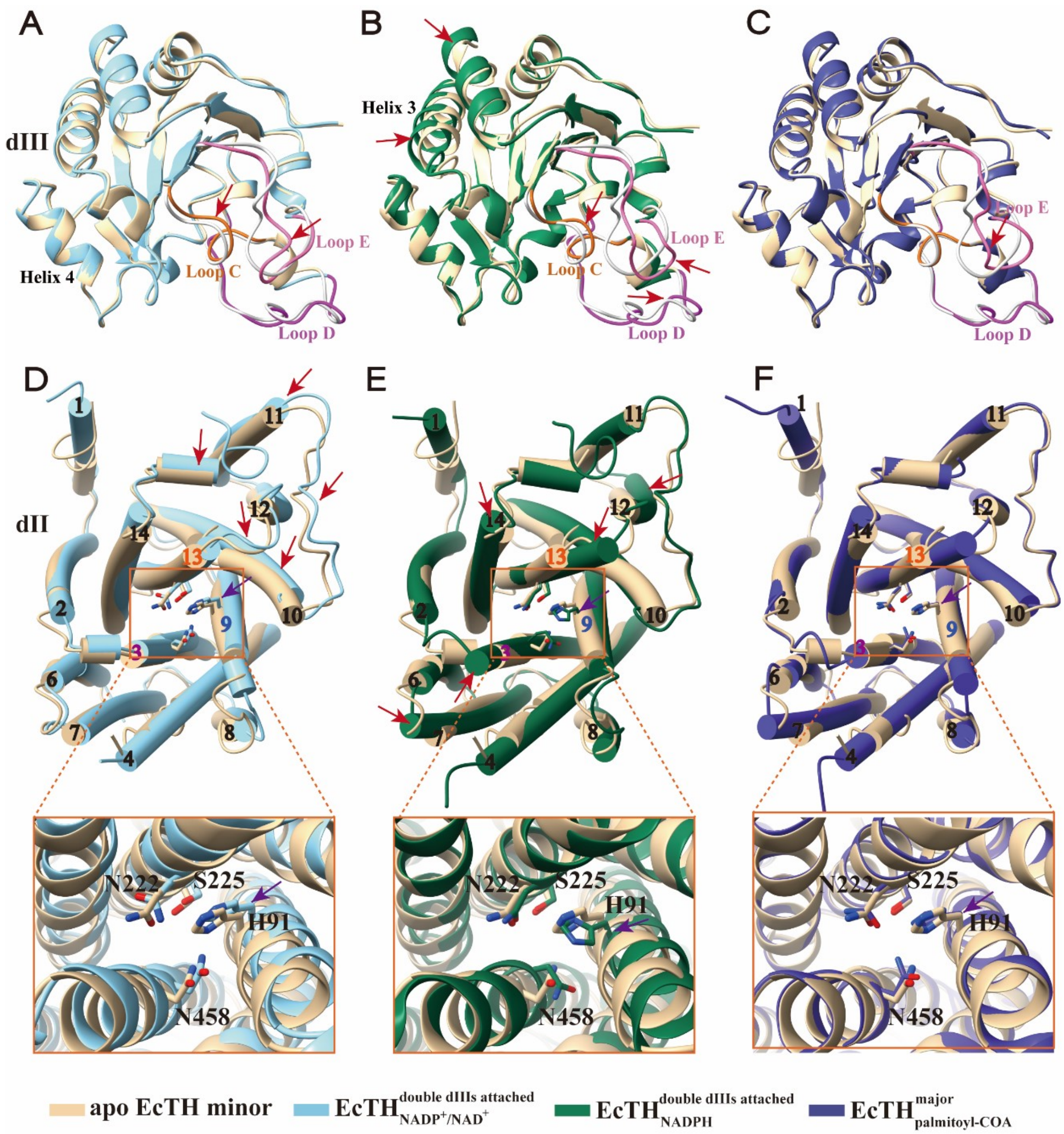

Fig. S9. NADP$^+$/NADPH binding to the dIII domain induces conformational changes in the proton translocation pathway and promotes opening of the NADP(H)-binding pocket. (A-C) Structural comparison of the face-down dIII domains in the NADP$^+$-bound (cyan), NADPH-bound (deep green), and palmitoyl-CoA-bound (Majorelle blue) states with the apo state (light brown). The dIII domains from $\mathrm{EcTH}^{\mathrm{double\ dIIIs\ attached}}_{\mathrm{NADP^+/NAD^+}}$, single-subunit $\mathrm{EcTH}^{\mathrm{double\ dIIIs\ attached}}_{\mathrm{NADPH}}$, and single-subunit $\mathrm{EcTH}^{\mathrm{major}}_{\mathrm{palmitoyl-CoA}}$ were individually aligned to the apo EcTH dIII using best-fit pairwise superposition. Red arrows indicate the regions showing significant displacement. Loops C, D, and E are colored chocolate, magenta and hot pink, respectively. NADP$^+$ binding induces conformational changes in these loops surrounding the NADP(H)-binding pocket, leading to pocket opening (Supplementary Video 1); NADPH binding produces more pronounced rearrangements in

both the surrounding loops and adjacent helices, resulting in enhanced opening with a distinct outward-displacement tendency (Supplementary Video 6). In contrast, binding of the inhibitor palmitoyl-CoA causes only minimal conformational changes in the surrounding loops and helices. (D-F) Corresponding comparisons of proton translocation pathways in the dII domains viewed from the cytoplasmic side, with cartoon colors matching those in panels (A-C). Alignment was performed using one side of the proton channel helices (TM3, TM9, and TM13). For additional details, see Figs. 2E, S7E and 4E.

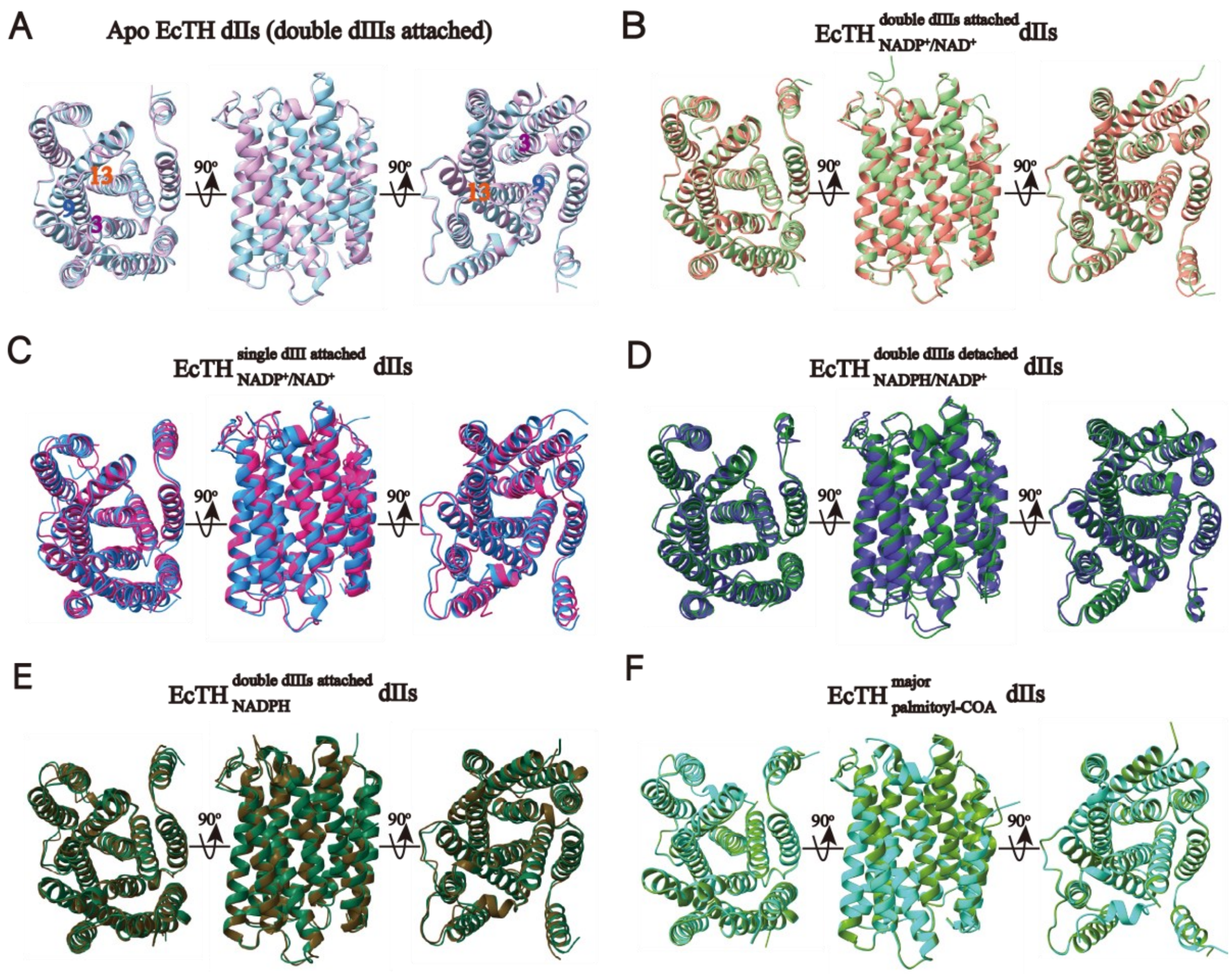

Fig. S10. Conformational comparison of bilateral proton translocation pathways in high-resolution dII domains across distinct states. (A-F) Structural comparisons of bilateral dII dimains from single-subunit apo EcTH, WT $\mathrm{EcTH}^{\text{double dIIIs attached}}_{\text{NADP}^+/\text{NAD}^+}$, WT $\mathrm{EcTH}^{\text{single dIII attached}}_{\text{NADP}^+/\text{NAD}^+}$, $\mathrm{EcTH}^{\text{double dIIIs detached}}_{\text{NADPH/NADP}^+}$, $\mathrm{EcTH}^{\text{double dIIIs attached}}_{\text{NADPH}}$ and single-subunit$\mathrm{EcTH}^{\text{major}}_{\text{palmitoyl}-\text{CoA}}$, viewed from the periplasmic (left), membrane (middle) and cytoplasmic (right) sides. In WT $\mathrm{EcTH}^{\text{single dIII attached}}_{\text{NADP}^+/\text{NAD}^+}$ (C), the dII domain on the dIII-detached side (deep pink cartoon) exhibits transmembrane helices displaced toward the cytoplasmic side, facilitating proton extrusion. In $\mathrm{EcTH}^{\text{double dIIIs detached}}_{\text{NADPH/NADP}^+}$ (D), structural asymmetry is observed between the two dII domains, shown in purple for the dII at the dI-dIII interface-forming side and in deep green for that at the non-interface-forming side, potentially arising from sequential dIII detachment. In the other conformations (A, B, E and F), the dIII domains remain largely symmetric.

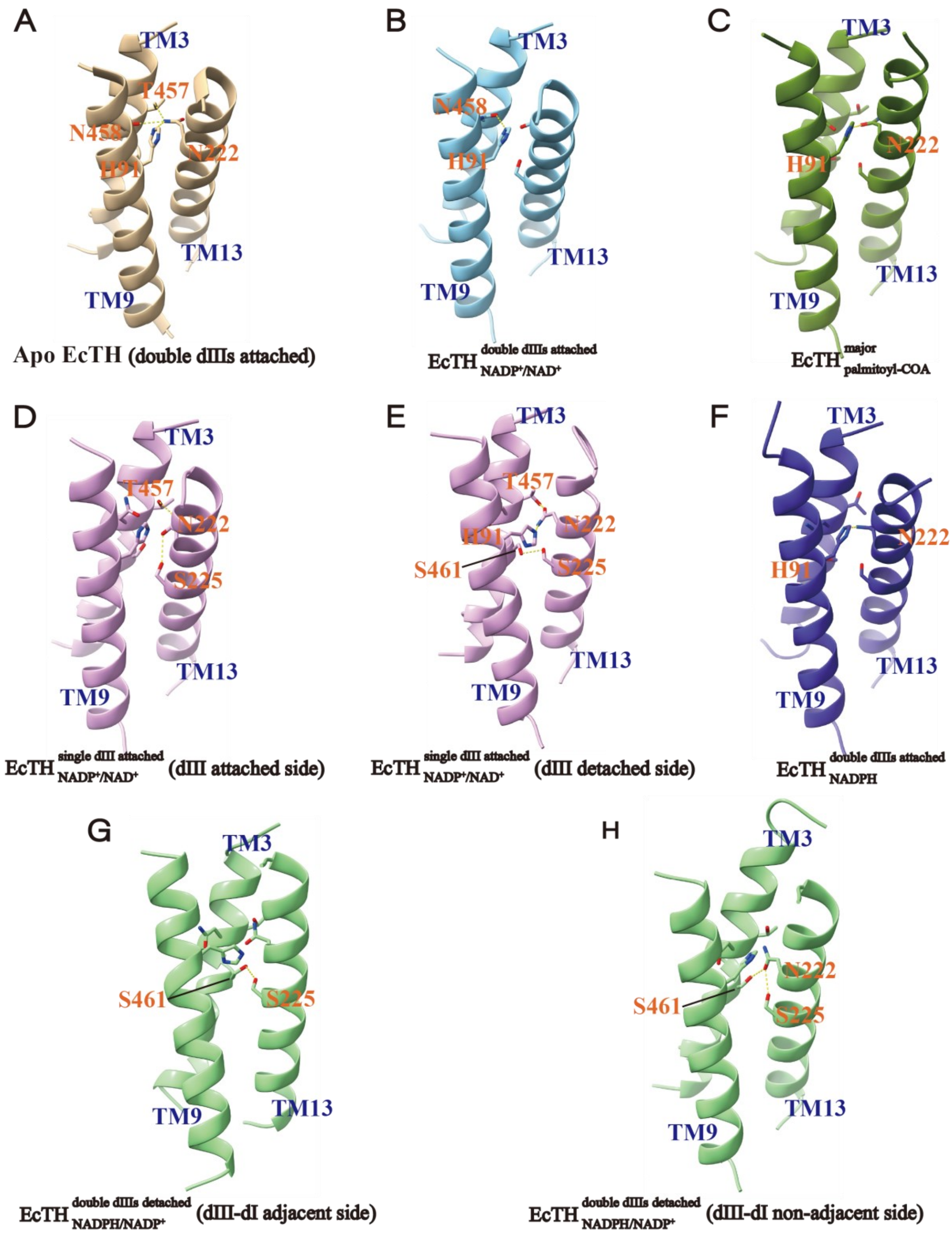

Fig. S11. Changes in residue positions and hydrogen bonds in the proton channel wall across different conformations. Across the various conformations, the proton-translocating residues, H91 on TM9, N222 and S225 on TM13, and S461, T457 and N458 on TM3 within the channel wall, are shown in cartoon representation. Residues involved in hydrogen bonding are labeled in orange, and unlabeled residues indicates that no

hydrogen bond was detected for that position. Hydrogen bonds are shown as yellow dashed lines.

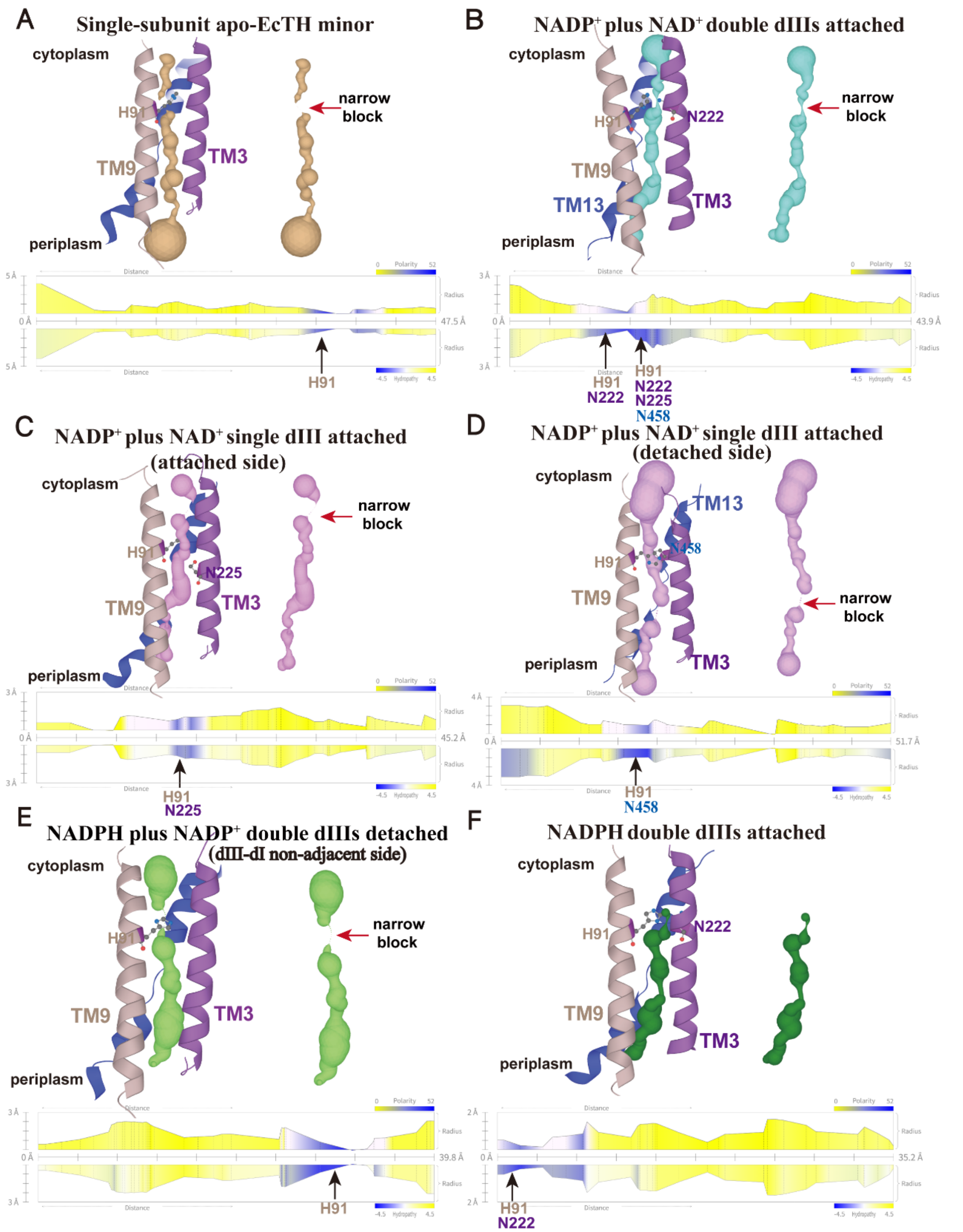

Fig. S12. Conformational dynamics of the proton-translocation channel. Structural features of the proton-translocating pore and its pathway profile (calculated using MOLE 2.5) between the N-side (cytoplasmic side) and the P-side (periplasmic side) are shown for the dII domains of (A) single-subunit apo EcTH, (B) WT $\mathrm{EcTH}^{\text{double dIIIs attached}}_{\mathrm{NADP^+/NAD^+}}$, (C) WT $\mathrm{EcTH}^{\text{single dIII attached}}_{\mathrm{NADP^+/NAD^+}}$ at dIII-attached side, (D) WT $\mathrm{EcTH}^{\text{single dIII attached}}_{\mathrm{NADP^+/NAD^+}}$ at dIII-

detached side, (E) $\text{EcTH}_{\text{NADPH/NADP}^+}^{\text{double dIIIs detached}}$ at the dIII-dI non-adjacent side, and (F) $\text{EcTH}_{\text{NADPH}}^{\text{double dIIIs attached}}$. The three helices TM3, TM9, and TM13 that constitutes the proton channel in domain II were selected as structural targets for channel prediction across these conformational states. For each conformation, the predicted proton-pathway profiles are illustrated using color-coded cartoon representations, shown both within the transmembrane helices and in isolation. Below each structural panel, the corresponding diameter profiles exported directly from MOLEonline are presented as quantitative plots. The horizontal axis denotes the amino-acid positions, and the vertical axis indicates the channel radius at each position. The upper color bar, ranging from yellow to blue, represents amino-acid polarity values ranging from 0 to 52, with blue highlighting polar residues predominantly localized around H91. The lower color bar, with a similar yellow-to-blue gradient, corresponds to amino acid hydropathy indices spanning from -4.5 to 4.5. In all views, the top and bottom of each helix correspond to the cytoplasmic and periplasmic sides, respectively.

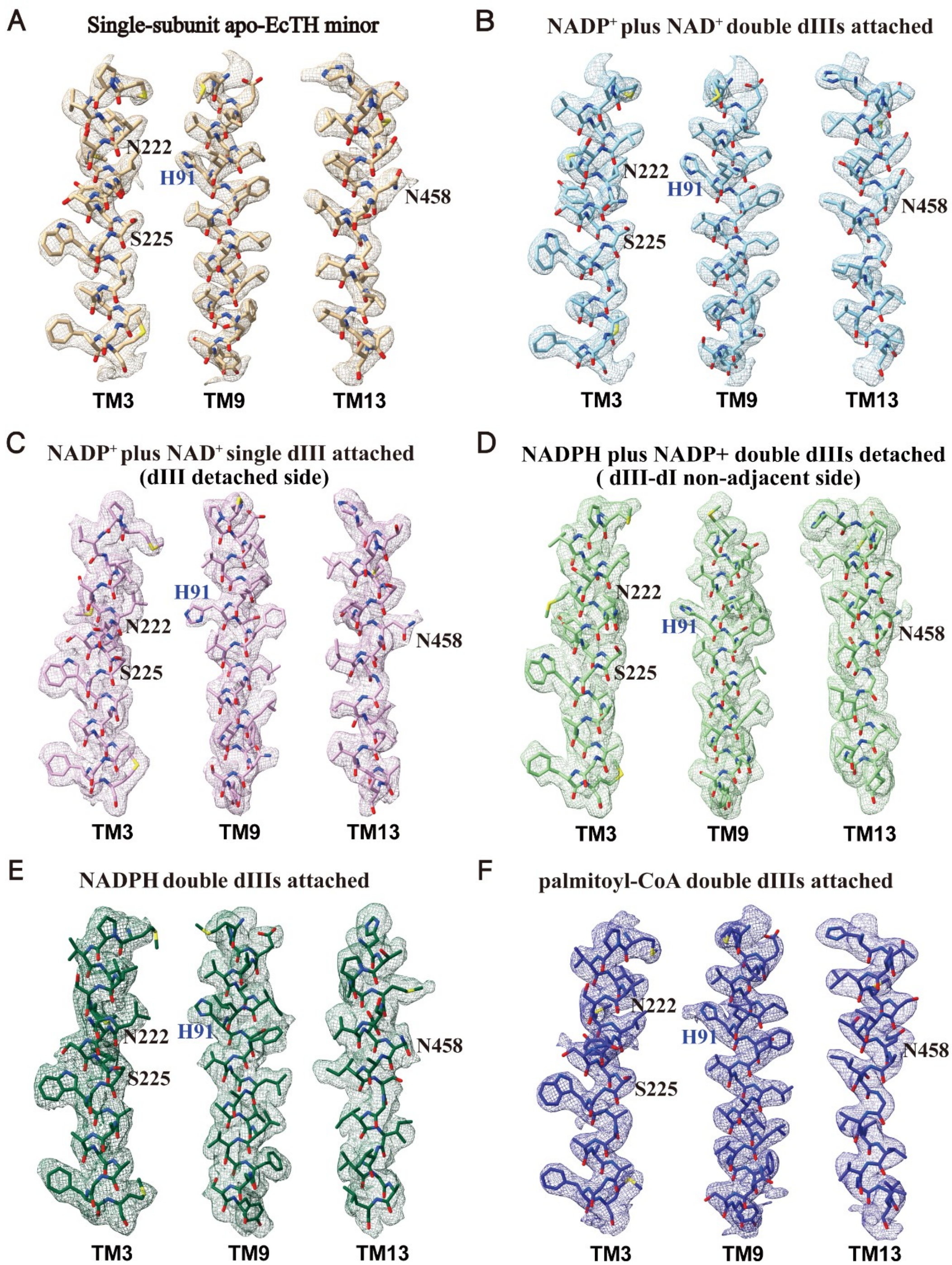

Fig. S13. Representative cryo-EM density maps of the transmembrane helices across different conformational states. Cryo-EM densities for TM3, TM9, and TM13 are shown for (A) single-subunit apo-EcTH (minor), (B) WT $\mathrm{EcTH}_{\mathrm{NADP^+/NAD^+}}^{\text{double dIIIs attached}}$, (C) dIII detached side of WT $\mathrm{EcTH}_{\mathrm{NADP^+/NAD^+}}^{\text{single dIII attached}}$, (D) dIII-dI non-adjacent side of $\mathrm{EcTH}_{\mathrm{NADPH/NADP^+}}^{\text{double dIIIs detached}}$, (E) $\mathrm{EcTH}_{\mathrm{NADPH}}^{\text{double dIIIs attached}}$ and (F) single-subunit $\mathrm{EcTH}_{\text{palmitoyl−CoA}}^{\text{major}}$, illustrating state-dependent changes in the dII proton channel.
